# Antifungal activity of Co(II) and Cu(II) complexes containing 1,3-bis(benzotriazol-1-yl)-propan-2-ol on the growth and virulence traits of fluconazole-resistant *Candida* species: synthesis, DFT calculations, and biological activity

**DOI:** 10.1186/s13065-023-01037-7

**Published:** 2023-10-10

**Authors:** Ricardo A. Murcia-Galán, Sandra M. Durán, Sandra M. Leal-Pinto, Martha V. Roa-Cordero, Jose D. Vargas, Laura V. Herrera, Alvaro Muñoz-Castro, Desmond MacLeod-Carey, Tonny W. Naranjo, Peter L. Rodríguez-Kessler, John J. Hurtado

**Affiliations:** 1 Grupo de Investigación en Química Inorgánica, Catálisis y Bioinorgánica, Departamento de Química, Universidad de los Andes, Carrera 1 No. 18A-12, 111711 Bogotá, Colombia; 2 Facultad de Ciencias Médicas y de la Salud, Universidad de Santander, Calle 70 No. 55-210, Bucaramanga, Colombia; 3 Grupo Sistema Estomatognático Y Morfofisiología (SEMF), Departamento de Ciencias Básicas, Universidad Santo Tomás Seccional Bucaramanga, Carrera 27 No. 180-395, Bucaramanga, Colombia; 4 Facultad de Ingeniería, Arquitectura y Diseño, Universidad San Sebastián, Bellavista 7, 8420524 Santiago, Chile; 5 Facultad de Ingeniería, Instituto de Ciencias Químicas Aplicadas, Inorganic Chemistry and Molecular Materials Center, Universidad Autónoma de Chile, El Llano Subercaseaux 2801, Santiago, Chile; 6 Experimental and Medical Micology Group, Corporación para Investigaciones Biológicas (CIB), 050010 Medellin, Colombia; 7 Facultad de Medicina, Universidad Pontificia Bolivariana, 050034 Medellín, Colombia; 8 Centro de Investigaciones en Óptica A.C., Loma del Bosque 115, Col. Lomas del Campestre, 37150 León, Guanajuato México

**Keywords:** Benzotriazole ligand, Cobalt(II) and copper(II) complexes, Antifungal activity, Antibiofilm agents, *Candida* species, Virulence traits

## Abstract

Relevant virulence traits in *Candida* spp. are associated with dimorphic change and biofilm formation, which became an important target to reduce antifungal resistance. In this work, Co(II) complexes containing a benzotriazole derivative ligand showed a promising capacity of reducing these virulence traits. These complexes exhibited higher antifungal activities than the free ligands against all the *Candida albicans* and non-*albicans* strains tested, where compounds **2** and **4** showed minimum inhibitory concentration values between 15.62 and 125 μg mL^−1^. Moreover, four complexes (**2**–**5**) of Co(II) and Cu(II) with benzotriazole ligand were synthesized. These compounds were obtained as air-stable solids and characterized by melting point, thermogravimetric analysis, infrared, Raman and ultraviolet/visible spectroscopy. The analysis of the characterization data allowed us to identify that all the complexes had 1:1 (M:L) stoichiometries. Additionally, Density Functional Theory calculations were carried out for **2** and **3** to propose a probable geometry of both compounds. The conformer **Da** of **2** was the most stable conformer according to the Energy Decomposition Analysis; while the conformers of **3** have a fluxional behavior in this analysis that did not allow us to determine the most probable conformer. These results provide an important platform for the design of new compounds with antifungal activities and the capacity to attack other target of relevance to reduce antimicrobial resistance.

**Supplementary Information:**

The online version contains supplementary material available at 10.1186/s13065-023-01037-7.

## Introduction

Antimicrobial resistance (AMR) occurs when bacteria, fungi, viruses, or parasites change over time and no longer respond to medicines used to treat infections, making them harder to treat, increasing the risk of disease spread, severe illness, and death. AMR is a primary concern for public health threats since a considerable number of strains of different pathogenic microorganisms with AMR are emerging, which has increased the morbidity and mortality rates of humans and animals due to infectious diseases [1, 2]. During 2019, nearly 4.95 million human deaths were associated with bacterial AMR, including 1.27 million deaths directly attributable to bacterial AMR [3].

This increase in AMR has occurred due to excessive and inappropriate use of antimicrobials and the transfer of resistant genes between homologous strains present in different environments (farm animals and the environment) [4, 5]. Moreover, infectious diseases of resistant strains primarily put at risk people undergoing surgical procedures, organ transplants, immunosuppressive procedures (such as chemotherapy), and the care of newborns [1, 6–11]. Among the resistant microorganisms, *Candida* strains stand out, of which several mechanisms linked to their resistance are already known: overexpression or mutation of the target enzyme, concordances of the biosynthetic pathway of the target compound, formation of biofilms, changes in the permeability of the cell membrane, among others [12–15]. The ability of *Candida* species to form biofilms makes it an important pathogen of infections associated with health care, and its presence on the surfaces of medical devices such as catheters is dependent on the ability of each species to produce extracellular polymeric substances (EPS) that contribute to the persistence of the microorganism [16]. Invasion and maintenance of biofilm architecture are further related to the ability of *Candida* to undergo a dimorphic change from yeast to hyphae, another important virulence trait [17]. For the above, the treatment of candidemia is a clinical challenge that demands the development of a new drug formulation with effects on virulence traits related to antifungal resistance. Thus, the inhibition of biofilm formation is an approach currently explored for developing antimicrobials that are more effective.

Compounds derived from azoles have been widely implemented against different strains of fungi due to their effectiveness and mechanism of action, which involves the inhibition of ergosterol synthesis that stops the growth and replication of the fungus [18, 19]. However, these azole compounds have decreased their antifungal activity due to the development of AMR, causing the cytotoxic effects of this type of medication to be at the same level as the antifungal effects and forcing the search for compounds that can continue implementing the mechanism of action of azoles but with minimal or less cytotoxicity.

In this sense, metal complexes or coordination compounds have recently aroused much interest in the scientific community, which generate changes in some chemical properties of the active compounds, also providing new possible routes of action, such as more effective delivery of active compounds [20–22]. These metal complexes have shown additional mechanisms of action, such as increased membrane penetration by increased lipophilicity, inhibition of the exchange of some enzymes with enzymatic ligands or substrates, and induction of oxidative stress by the generation of reactive oxygen species, among others [23–29]. These enhanced characteristics possessed by metal complexes make them potential candidates for new compounds with antimicrobial activity that could prevent or reduce the problem of antimicrobial resistance. In addition to the requirement that the metal centers possess potential antimicrobial activity, they must be biocompatible and bioavailable, where copper and cobalt stand out [30, 31].

Computational calculations based on density functional theory (DFT) have played an important role in recent years, supporting the structural study of coordination compounds, as well as their antimicrobial activities. From these calculations, it has been possible to evaluate the chemical reactivity of these compounds, as well as to identify their physicochemical and electronic characteristics and properties [32–34]. By carrying out these computational studies, it has been possible to establish the possible geometries of the coordination compounds, the distribution of electronic densities throughout the complex, the energetic differences between the molecular orbitals (frontier molecular orbitals analysis) and to elucidate the possible interactions of those with different cell areas (enzymes, cell wall and membrane, among others).

Herein, we report the synthesis and characterization of four new coordination complexes containing Co(II) and Cu(II) as metal centers and ligands derived from benzotriazole and we tested the bioactivity on fluconazole-resistant isolates of *Candida* species and reference strains, epidemiologically relevant. This aim was achieved by exploring the effect of these metal complexes on planktonic growth and two major virulence traits as well as yeast-to-hyphae transition and biofilm growth. In addition, we assayed the antiproliferative activity in murine macrophages to know the selectivity of the coordination complexes synthesized here. Such assays are pivotal to characterize and estimate the promissory value of these molecules for further development of inorganic medicinal chemistry targeting biologically relevant and versatile metallic complexes.

## Results and discussion

### Synthesis of 1,3-bis(benzotriazol-1-yl)-propan-2-ol (1)

Several tests were performed using the previously reported methods from literature [35], though it was impossible to duplicate the synthetic procedure to obtain **1**. As a result, this article presents an alternative synthesis route consisting of a phase-transfer catalyzed coupling reaction between 1,3-dichloro-propan-2-ol and 1*H*-benzotriazol followed by thin-layer chromatography (Additional file 1: Figure S42).

Compared to the work reported by Zhang *et* *al*. the product was separated as a white, air-stable solid with a higher yield. Elemental analysis, FT-IR, Raman spectroscopy, ^1^H, and ^13^C NMR were used to characterize **1**, allowing for a highly pure identification of the ligand.

### Synthesis and characterization of metal complexes

**Fig. 1:**
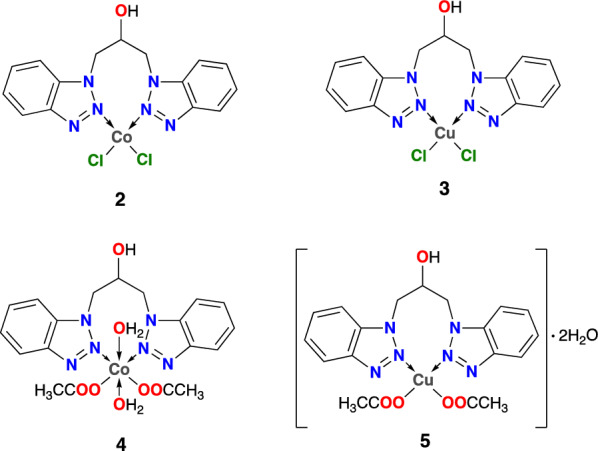
Possible structures of the complexes under study

The complexes were synthesized using either a pure solvent or a mixture of solvents that could dissolve both the ligand and the corresponding metallic salts. In order to produce **2** and **3**, the reaction was carried out in acetone, where immediate precipitation of the complexes was observed. This solvent was also used to wash the end products. Due to the polar protic solvent affinity for metallic salts in cases **4** and **5**, a solvent mixture was required. Tetrahydrofuran and methanol form the solvent mixture, where the product is kept in solution and removing the solvent at the end of the reaction is necessary. All the complexes (**2**–**5**) were isolated as non-hygroscopic and air-stable solids. Figure 1 shows the proposed structures of the complexes under investigation.

Furthermore, **2**–**5** only show significant solubility in polar protic solvents such as ethanol and methanol. Elemental analysis of the complexes revealed that **5** possess hydration water molecules and **4** likely had water molecules apparently coordinated to the metal core. These statements are supported by thermal analysis.

#### FTIR spectroscopy

All complexes were studied using infrared spectroscopy (FT-IR) to determine the shifts of specific bands associated with ligand stretching molecular vibrations upon coordination with the metal core. Table 1 provides a summary of the assignment of important bands in the ligand and its complexes.

**Table 1:** Infrared spectral bands for ligand **1** and its complexes

Compound	Wavenumber ν /cm^−1^						
	(O–H)	(C-H)	(C-H)	(C = O)	(N–N)	(C-N)	(C-O)
**1**	3368 s	3067 w	2924 w	–	1454 m	1227 vs	1092 s
**2**	3491 m	3098 w	2928 w	–	1458 m	1231 vs	1026 s
**3**	3379 m	–	–	–	1458 m	1234 s	1092 m
**4**	3368 s	3067 w	2924 w	1558 vs	1415 vs	1227 s	1096 m
**5**	3367 m	3067 w	2920 w	1609 vs	1423 vs	1227 s	1084 m

*w: weak; m: medium; s: strong; vs: very strong

Relevant groups in the structure of the free ligand (**1**) exhibit distinctive infrared bands. Two stretching bands observed in the FT-IR spectra were related to the alcohol group, with bands at 3368 cm^−1^ and 1092 cm^−1^ assigned to O-H and C-O bonds, respectively. The C-H stretching band found at 3067 cm^−1^ was assigned to the aromatic system (found in the benzotriazole moiety), and the band at 2924 cm^−1^ was assigned to aliphatic C-H stretching corresponding to the propan-2-ol spacer. Finally, peaks at 1454 cm^−1^ and 1227 cm^−1^ were assigned to N–N and C-N stretching vibration of the benzotriazole fragment. The coordination of the ligand to a metal center can be confirmed using these peaks most effectively [36, 37].

The metal coordination to the nitrogen of the azole group in **2** and **3** is explained by a shift in the N–N and C-N bands with respect to **1** [38, 39]. Thus, the hypothesized coordination mode in Fig. 1 is validated. While the C-N bond band shifts to 1231 and 1234 cm^−1^ for complexes **2** and **3**, respectively, the N–N bond band shifts to 1458 cm^−1^ for both complexes. In the case of **4** and **5**, the C-N bond band does not shift from its position in **1**. However, a larger shift in the band assigned to N–N bond was observed for **4** and **5**, shifting to lower wavenumbers (1415 and 1423 cm^−1^, respectively). Raman spectroscopy will be used to explain this behavior. However, due to the presence of acetate ligands, compounds **4** and **5** present a new band at 1558 and 1609 cm^−1^ that was attributed to the stretching of the C=O bond. The M-OH_2_ stretching band was expected to be visible between 1600 and 1630 cm^−1^ [40], but it is masked by bands for the acetate ligand. Raman spectroscopy could be used to observe these bands.

#### Raman spectroscopy

Compounds **1**–**5** were analyzed by Raman spectroscopy to contribute to their characterization. Table 2 summarizes the assignment of the selected bands in the spectra of **1**–**5**.

**Table 2:** Raman spectral bands for **1** and its complexes

Wavenumber/cm^−1^					
Assignment	Compound				
	**1**	**2**	**3**	**4**	**5**
ν (M–O_(acetate)_)	–	–	–	74	116
ν (M-Cl)	–	343	375	–	–
δ (triazole ring)	542	539	544	–	542
δ (triazole ring)	620	620	619	615	625
δ (M-L)	–	951	937	930	938
ν (N–N-N triazole ring)	1165	–	1170	1166	1165
δ (triazole ring)	1233	1226	1233	1227	1229
ν (triazole ring)	1379	1388	1373	1385	1379
δ (benzene ring)	1498	1494	1490	1488	1495
δ (benzene ring)	1590	1599	1589	1586	1590
ν (O–H)	3369	–	3448	3367	3335

The bands over 3300 cm^−1^ in the ligand and complexes were assigned to the O–H stretching from the propan-2-ol moiety of the ligand. For **1**, bands observed at 1379, 1233, 620, and 542 cm^−1^ were assigned to the triazole ring stretching, breathing, bending, and torsion, respectively [41–44]. Another band of interest is located at 1165 cm^−1^, which is assigned to the N–N-N symmetric stretching [44, 45]. As a result of the coordination of the metal to the N of the benzotriazole, these bands appear to shift in the complex spectra, suggesting a reduction in the rigidity of the triazole ring. The bands observed at 343 cm^−1^ (**2**) and 375 cm^−1^ (**3**) are assigned to the Co-Cl and Cu-Cl vibration, respectively [46, 47]. While bands observed at 74 cm^−1^ (**4**) and 116 cm^−1^ (**5**) are associated with the bond between the metal center and the oxygen from the acetate co-ligand [48].

Additionally, the bands at 951 cm^−1^ (**2**), 937 cm^−1^ (**3**), 930 cm^−1^ (**4**), and 938 cm^−1^ (**5**) were only observed in the complexes spectra and may be associated with the different interactions between cobalt and copper center and the ligand. Finally, the stretching bands at 1590 and 1498 cm^−1^ in the ligand associated with the benzene ring [41, 43, 45], shifted toward lower wavenumbers in the spectra of the complexes, denoting a decrease in the rigidity of this ring.

#### UV/Vis spectroscopy

To study electronic properties related to the complex characteristics, the UV–Vis spectra of the compounds were recorded in methanol. The bands for the ligand, complexes **2**–**5**, and metallic salts used for complexation, are shown in Table 3**.**

**Table 3:** Excitation bands for the starting metallic salts, ligand (**1**) and complexes (**2**–**5**) were observed in their UV/Vis spectra

Compound	Transition (nm) ($\varepsilon$(M^−1^ cm^−1^)					
	UV			Vis		
	${\lambda }_{1}$	${\lambda }_{2}$	${\lambda }_{3}$	${\lambda }_{1}$	${\lambda }_{2}$	${\lambda }_{3}$
**1**	204 (76,272)	262 (23,597)	280 (16,771)			
**2**	204 (39,461)	263 (12,404)	280 (9304)	530 (25)		
**3**	203 (60,623)	262 (17,372)	276 (11,714)	860 (106)		
**4**	203 (32,949)	261 (10,264)	280 (8426)	514 (28)		
**5**	204 (49,112)	262 (13,205)	278 (9498)	424 (192)	450 (262)	714 (38)
CoCl_2_·6H_2_O	202 (688)	268 (107)		522 (18)		
CuCl_2_·2H_2_O	208 (6142)	270 (8163)		896 (66)		
Co(CH_3_COO)_2_·4H_2_O	204 (1031)	268 (47)		518 (14)		
Cu(CH_3_COO)_2_·H_2_O	246 (4269)			698 (94)		

All compounds (**1–5**) exhibit three bands between 200 and 300 nm, which correspond to $\pi \to {\pi }^{*}$ orbitals electronic transitions of the ligand. These very intense bands, which merely changed in intensity, do not exhibit a relevant wavelength shift in the complexes spectra compared to the free ligand spectrum. While cobalt(II) complexes (**2**, **4**) exhibit a blue color in solution and pink color as a solid, copper(II) complexes (**3**, **5**) have a green hue in both the solid and solution phases.

Cobalt(II) salts and their complexes (**2**, **4**) only exhibit one band at 510–530 nm, which could be assigned to the ^*4*^*T*_*1g*_* (F)* $\leftarrow$ ^*4*^*T*_*1g*_ *(P)* transitions in a distorted octahedral geometry in solution [49, 50]. Bands at 420–900 nm were observed in the spectra of copper(II) salts and their complexes (**3**, **5**), which may be assigned to the single possible transition of the *d*^*9*^ metal [49, 51]. However, it cannot be specifically attributed to any particular geometry. A bathochromic shift in the bands in the visible region was identified when comparing the spectra of the complexes (**2**–**5**) with their respective salts.

#### Thermal analysis

The suggested TG and DTG findings are based on the expected mass losses for all complexes. The stages of decomposition, temperature ranges, and decomposition products, as well as the weight loss percentages of the complexes, are given in Additional file 1: Table S1, all the TGA and DTG data are shown in the Additional file 1.

Upon thermal decomposition, complex **3** produces a final residue containing metal and chlorine, whereas carbon and oxygen are also present in the end residue of complex **2**. A final residue containing metal, carbon, and oxygen was encountered for the other complexes [52–54]. Furthermore, the thermograms show a partial decomposition above 600 ºC.

In the case of complex **2**, a weight reduction of 31.63% was observed between 152 and 352 ºC, which implies a loss of a fragment of **1** corresponding to one benzotriazole plus one CH_4_ equivalent. The decompositions of the remaining benzotriazole fragment and one CH_2_ equivalent occurring as three weight loss processes (31.91%) were observed (31.16% calculated). These weight losses leave a metallic residue containing the salt with carbon and oxygen. On the other hand, **3**, suffers a slight weight loss between 27 and 170 ºC, which was only possible to link with a small amount of molecular hydrogen. The second loss of 9.25% was observed and assigned to a partial propane fragment. Over 253 ºC, a major reduction in weight of 62.14%, was attributed to the loss of two benzotriazole fragments with some additional atoms. Finally, a 3.40% weight drop occurs, leaving a metallic residue.

The thermogram of **4**, shows an initial weight loss between 27 and 239 ºC (with the peak temperature for the decomposition stage at 159 ºC), which may be related to the loss of a carbon atom and two water molecules coordinated to the metal center, both of which needed high temperatures to be released. The loss of ligand (**1**) is attributed to two decomposition stages that occur at 239–358 and 358-573 ºC, with weight losses of 34.10 and 22.81%, respectively. A final weight reduction takes place, leaving a metallic residue with carbon and oxygen, possibly corresponding to a metallic oxide with organometallic features [52–54].

For complex **5**, there is an initial loss that may be related to the removal of two coordination water molecules, similar to complex **4**. Additionally, the loss of the majority of the ligand (**1**) was connected to the second and third decomposition stages with weight reductions of 25.35 and 28.37%, respectively. Finally, a weight reduction of 8.80% is associated with the propan-2-ol fragment, which results in a metallic residue [52–54] similar to the one of **4**. The complexes containing chloride ligands are more stable than those containing acetate ligands. This is supported by the fact that the observed main weight losses of **2** and **3** have a larger maximum DTG and TG range compared to **4** and **5**.

### Biological studies

#### Effect on planktonic cells

Co(II) and Cu(II) complexes containing 1,3-bis(benzotriazol-1-yl)-propan-2-ol, ligand, and its salts were evaluated against *Candida* spp. and mammalian cells. The results of MIC and CFM are detailed in Additional file 1: Table S2.

The ligand (**1**), Cu(II) salts, and Cu(II) complexes (**3** and **5**) did not show a significant inhibitory effect against any of the *Candida* strains evaluated (MIC > 1000 μg mL^−1^). The Cu(II) center has a dual role, and it is an essential micronutrient to fungal growth and proliferation but also exhibits antimicrobial properties due to the generation of reactive oxygen species [55], which are exploited by the immune cells to kill pathogens through increasing and/or limiting copper levels during infection. *C. albicans* copes with elevated or decreased concentrations of Cu by different mechanisms, such as (1) swapping metal cofactors of superoxide dismutase and increases in expression of transporter CTR1 for Cu uptake [56]; (2) stimulating the expression of copper-transporting P-type ATPase to increase the Cu efflux and 3) chelating copper by expression of metallothioneins [57]. One of these three mechanisms could explain the resistance exhibited by *Candida* strains to copper salts and complexes **3** and **5** tested here.

On the contrary, cobalt(II) salts and their complexes (**2** and **4**) significantly inhibited planktonic cells of *Candida* spp. (MIC 7.81–125 μg mL^−1^). The antifungal effect of complex **4** on *C. albicans*, *C. tropicalis* and *C. parapsilosis* (reference strain and clinical isolate, MIC 62.5 μg mL^−1^) was similar; however, *C. glabrata* MYA2950 showed greater sensitivity to both **2** and **4**, compared to the CAPF-07 isolate. Likewise, complex **2** exhibited a greater inhibitory effect against fluconazole resistance *C. tropicalis* isolate than its ATCC counterpart (Additional file 1: Table S2). Complex **4** shows better MIC against all *Candida* strains, due to the presence of co-ligand acetate in its structure, which could inhibit biosynthesis pathways of some aminoacids, generate stress in intracellular pH homeostasis, and inhibit the activity of efflux pumps [58, 59]. Importantly, the antifungal activity of the complexes was > 8 × greater than the free ligand. The obtained results were compared with the standard drugs FCZ and ITZ according to CLSI protocol.

Most of the studies that evaluate the minimum inhibitory concentration of coordination complexes, test their activity against *Candida albicans*, where it has been found that Co(II) compounds with ligands that contain azoles in their structure present MIC values between 12.5 and 400 μg mL^−1^ [60], 31.25 and 62.5 μg mL^−1^ [61], and 7.8–15.6 μg mL^−1^ [62], showing values close to those obtained with the complexes presented in this work. However, it has been found that these compounds become significantly cytotoxic, and they have even evaluated the possible cause of this effect [62]. Other articles have studied both Co(II) and Cu(II) complexes, presenting the same MIC values against *C. albicans*, 125 μg mL^−1^ [62]. When evaluating the cytotoxicity of Cu(II) complexes, it was found that the concentration at which it is not cytotoxic no longer presents antibiofilm activity [63].

Those studies that have evaluated the antifungal activity against different strains of *Candida* (*C. albicans*, *C. tropicalis, C. parapsilosis, C. glabrata, and C. krusei*), of Co(II) and Cu(II) complexes with azole derivative ligands, have presented MIC values of 15.62–100 μg mL^−1^ (Co complexes) [64], 1.75–50 μg mL^−1^ (Cu complex) [65], and 31.25–250 μg mL^−1^ (Co complexes) [66]. In addition, these complexes showed greater activity against *Candida* non*-albicans* strains, like what was observed with the complexes obtained in this study. In addition, in the present study, it is also observed that the MIC values of the Co(II) complexes present the same activity against sensitive and resistant strains. Several literature reports use the disk diffusion method to test the antifungal activity [67–72], which does not allow comparison with the results presented here. In addition, most articles only carry out biological activity tests against *C. albicans*. In other studies, Co(II) and Cu(II) complexes with ligands derived from isatin and sulfonamides have been reported, in which it has been observed that these present percentages of inhibition between 41 and 77% against strains of *Candida albicans* and *Candida glabrata*, with Co(II) complexes being more active than Cu(II) complexes with both types of ligands [73, 74].

#### Drug interactions

Interactions between bioactive cobalt(II) complexes (**2** and** 4**) and reference drugs (fluconazole and caspofungin) were evaluated on planktonic cells of *C. albicans* and *C. tropicalis* (ATCC strains and clinical isolates). We prioritize these species because they were less susceptible to complexes effects than other strains assayed here. Moreover, they are more prevalent in clinical cases of Candidemia [75]. Indifferent and additive interactions were observed in all the strains studied. However, combinations of MICs of caspofungin and complex **4** were synergistic against *C. tropicalis* 66,029 (FIC caspofungin 0.09 versus FIC complex **4** 0.25; ∑FIC Index 0.34). This combination allowed for a four-fold decrease of the MIC values of complex **4** (62.5 μg mL^−1^ to 15.62 μg mL^−1^) and caspofungin (0.125 μg mL^−1^ to 0.031 μg mL^−1^). Additionally, an antagonistic effect was observed on *C. albicans* 90,028 when the MICs of FCZ and complex **4** were combined (FIC FCZ 0.15; FIC C3 6; ∑FIC Index 6.15). The isobolograms of the synergistic and antagonistic combinations are shown in Additional file 1: Figure S41. This finding of synergistic interaction is interesting, since combined therapies could be more effective and less toxic than monotherapies. Explorations of the toxic effect of this combination on mammalian cells must be addressed in future studies to answer if the selectivity index improves.

#### Effect on virulence traits of *Candida* spp.

**Fig. 2:**
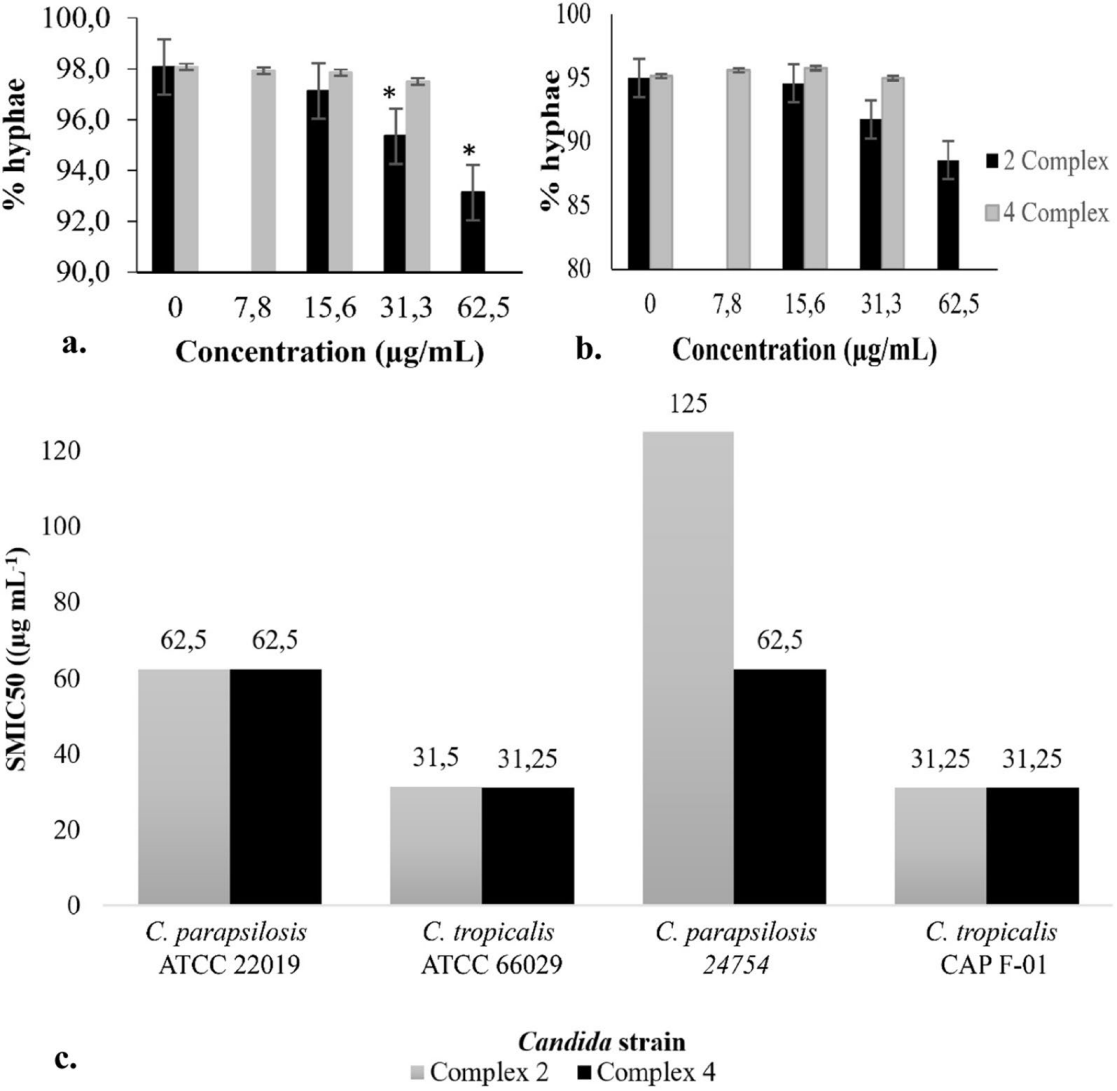
Inhibition of filamentation by Co(II) complexes (**2**, **4**). Blastospores treated with or without different concentrations of **2** and **4** were incubated in the hyphae-inducing medium at 37 °C, for 4 h. Then, the percent of filamentation and the percent of inhibition was calculated. Bars represent standard error (S.E.). Complexes **2** and **4** inhibited the *C. albicans* ATCC 90028 filamentation in 2.8 and 5%, respectively (**a**), while *C. albicans* CAP F-13 hyphal growth was inhibited in 3.4 and 7.8%, respectively (**b**). *P < 0.05. Anti-biofilm activity. Sessile minimum inhibitory concentration 50 (SMIC_50_) of cobalt (II) complexes **2** and **4** on biofilm of *Candida* spp (**c**)

The effect of the cobalt(II) complexes was studied against biofilm and hyphal morphogenesis, two major virulence phenotypes of *Candida* spp, see Fig. 2. The dimorphic switch from yeast to hyphae is considered the transition from the commensal to the pathogenic lifestyle of *C. albicans* [75]. However, yeast and hyphae have different roles during infections. In this way, hyphal growth is an important morphotype to penetration and invasiveness, while the yeast form is related to dissemination [76]. In the present study, we observed that subinhibitory concentrations of complex **2** (31.3 and 62.5 µg/mL) significantly reduced the dimorphic switch in *C. albicans*, while complex **4** did not affect the hyphae growth (Fig. 2a). The dimorphic switch is also related to biofilm biogenesis. The biofilms are a physical and metabolic barrier that *Candida* uses to establish infection, grow on different surfaces (tissue and medical devices), and resist antifungal therapy [77]. Such results indicate that complexes **2** and **4** have an inhibitory effect against established biofilms of ATCC strains and clinical isolates of *C. tropicalis* and *C. parapsilosis* after 24 h of incubation with SMIC_50_ from 31.25 to 62.5 μg mL^−1^. In contrast, this effect was not observed with the biofilm of *C. albicans* and *C. glabrata* (SMIC_50_ 500- > 2000 μg mL^−1^), probably due to the differential composition of the biofilm matrix depending on each strain (see Fig. 2b) [17]. Likewise, these results could be explained by the mild suppression (2.8–7.8% of inhibition) of hyphae morphogenesis that was observed in blastospores of *C. albicans* treated with these complexes. Interestingly, *C. tropicalis* biofilm showed higher susceptibility to cobalt complexes independent of the precursor salt used in the synthesis. These results are significant considering that there are no studies reporting the anti-biofilm effect of Co(II) complexes containing 1,3-bis(benzotriazol-1-yl)-propan-2-ol against strains of *Candida* spp. resistant to fluconazole.

#### Cytotoxicity in vitro

**Fig. 3:**
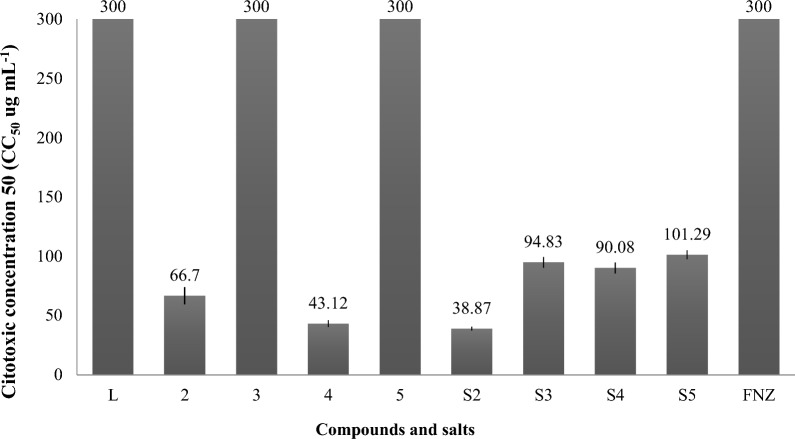
Cytotoxicity in macrophages J774.A1 of Co(II) and Cu(II) complexes containing 1,3 bis(benzotriazol-1-yl)-propan-2-ol. **L**: ligand (**1**); S2: CoCl_2_⋅6H_2_O; S3: CuCl_2_⋅2H_2_O; S4: Co(CH_3_COO)_2_⋅4H_2_O; S5: Cu(CH_3_COO)_2_⋅H_2_O; FNZ: fluconazole

The results are shown in Fig. 3. The ligand, copper(II) complexes (**3, 5**) and its salts did not show to be cytotoxic on murine macrophages (CC_50_ > 90 µg mL^−1^). However, cobalt(II) complexes (**2**, **4**) bioactive on *Candida* spp. and cobalt(II) salt CoCl_2_⋅6H_2_O were partially toxic (CC_50_ 38.87 – 66.7 µg mL^−1^) with SI between 0.93 -1.44. In the case of complex **2**, the union of the ligand and the salt allowed a decrease of almost twice the CC_50_, contrary to the complexation to form complex **4**, where it was shown to affect the cellular viability in comparison with the salt alone.

### DFT calculations

Density functional theory (DFT) calculations were employed in a computational study for complexes **2** and **3**. While **3**, which has a d^9^ metal core, only has one conceivable electronic distribution (D, a doublet), **2**, which has a d^7^ metal center, has two possible electronic distributions, with either one (D, a doublet) or three (Q, a quadruplet) unpaired electrons. Four distinct conformers were also investigated, considering the different multiplicities of each complex.

The relative bonding energies of the different conformers (a–d) of both complexes at their different electronic distributions at their optimized structures are shown in Table 4. It can be noted that the differences between the different conformers are negligible based on these energies. According to bonding energies, in the case of **2**, the most stable conformers are those with three unpaired electrons. However, the encountered differences do not surpass 1 eV. The structures of the conformers are presented as Additional file 1.

**Table 4:** Relative bond energy of the conformers (a-d) of complexes **2** and **3**, considering different electronic distributions (D = doublet, Q = quadruplet)

Complex/Multiplicity/Conformer		Relative bonding energy (eV)
**2**	Da	0.28
	Db	0.00
	Dc	0.17
	Dd	0.29
	Qa	0.37
	Qb	0.05
	Qc	0.00
	Qd	0.05
**3**	Da	0.31
	Db	0.00
	Dc	0.21
	Dd	0.42

The conformers of **2** and** 3**, in a doublet electronic configuration present distorted tetrahedral and square planar geometries, whereas the conformers in a quadruplet electronic configuration only present a distorted tetrahedral geometry. The structural features that give rise to the different conformations considering the bond angles between the metal center and the coordinated chlorine and nitrogen atoms are presented in Table 5. It is observed that the chlorine atoms had greater mobility in comparison to the nitrogen’s, whose movement is restricted by the rigidity of the ligand **1**. The conformers of **2** in a doublet and quadruplet electronic configuration have similar or equal bond lengths between Co center and nitrogen of the ligand, due to the restricted mobility of nitrogen in the ligand; however, the bond length between Co center and each chlorine has a significant difference, 5–8 pm. In these conformers, it is observed that the bond length of the Co–N is lower when the geometry of the conformer is close to the square planar and increases by being closer to the tetrahedral. For the conformers of **3**, it is observed a behavior like the conformers of complex **2**, except for **3Da**; this has different bond lengths for each Cu–N, which is related to the tetrahedral geometry that is more distorted than for others.

**Table 5:** Bond angles and bond lengths of the conformers of **2** and **3**

Complex/Multiplicity/Conformer		Bond angle (º) and Bond length (pm)					
		N_x_-M-Cl_a_	N_x_-M-Cl_b_	N_y_-M-Cl_a_	N_y_-M-Cl_b_	N-M-N	Cl-M-Cl
**2**	Da	93.6	130.8	119.5	100.7	104.6	109.5
		Co-Cl	223.1	217.8	Co-N	200.2	202.6
	Db	117.1	110.3	117.1	110.3	86.8	112.7
		Co-Cl	217.5	223.9	Co-N	205.3	205.3
	Dc	91.8	94.4	91.8	94.4	133.8	164.2
		Co-Cl	219.4	224.7	Co-N	196.4	196.4
	Dd	92.4	90.8	92.4	90.8	157.7	162.8
		Co-Cl	223.7	219.2	Co-N	190.1	190.1
	Qa	102.2	109.8	108.7	107.1	107.2	121.1
		Co-Cl	223.6	218.9	Co-N	207.7	207.4
	Qb	114.3	111.4	114.3	111.4	87.2	115.2
		Co-Cl	218.3	225.9	Co-N	206.9	206.9
	Qc	114.4	111.5	114.4	111.5	86.6	115.3
		Co-Cl	218.0	226.2	Co-N	207.9	207.9
	Qd	111.1	114.1	111.1	114.1	87.1	115.8
		Co-Cl	225.9	218.2	Co-N	207.4	207.4
**3**	Da	93.0	143.2	125.3	101.7	94.1	104.1
		Cu-Cl	225.4	218.6	Cu-N	213.8	230.6
	Db	125.7	106.6	125.7	106.6	79.1	109.1
		Cu-Cl	217.3	225.5	Cu-N	225.0	225.0
	Dc	91.1	93.1	91.1	93.1	139.4	168.0
		Cu-Cl	223.3	227.5	Cu-N	213.7	213.7
	Dd	94.8	95.6	94.8	95.6	131.1	154.6
		Cu-Cl	228.8	222.3	Cu-N	212.8	212.8

**Fig. 4:**
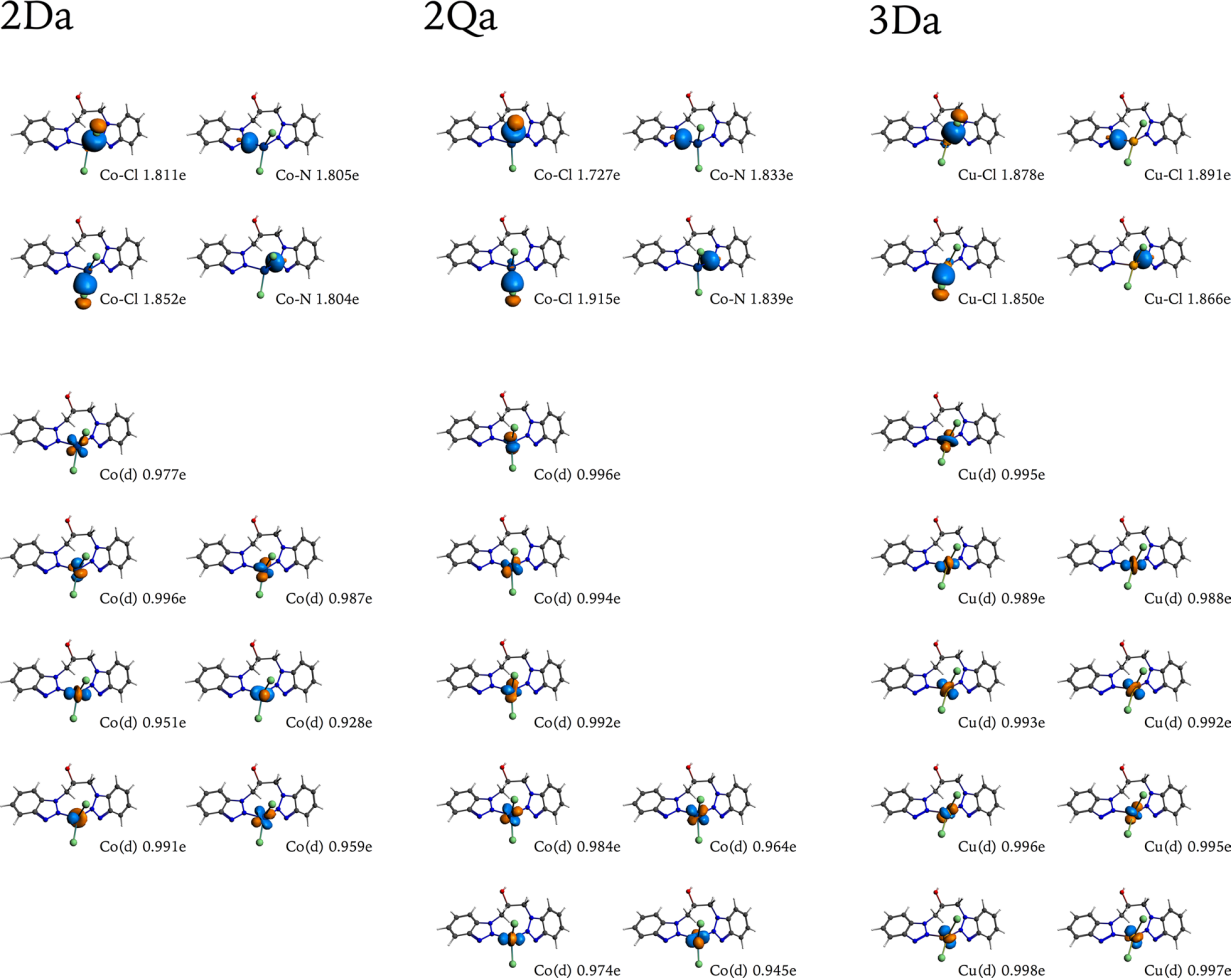
Selected NBO with their respective occupation numbers for **2Da**, **2Qa**, and **3Da**, represented using a cutoff isosurface value of 0.070

With the aim of gaining a deeper understanding of the electronic structure of these complexes, a Natural Bond Orbital (NBO) analysis was performed. The natural population analysis (NPA) gives the population of the natural localized atomic charges for the studied systems at their different electronic states, whose results are presented in Fig. 4. The four top isosurface plots represent the bonding interaction of the respective ligands, with the total corresponding occupation number including the spin α and spin β contributions. In all cases the occupation can be approximated to 2 e, as is expected for a Chloride (Cl^–^) anionic donating and lone-donating pairs from nitrogenated (N:) ligands. The bottom isosurface plots represent the unpaired electrons from the metal atoms separated as spin α and spin β. In the case of **2Da** and **2Qa**, a d^7^ electron configuration is expected for Co^2+^, with one unpaired spin α electron for **2Da** and three unpaired spin α electrons for **2Qa**, see Fig. 4. In the case of the copper complex, a d^9^ electron configuration is expected for Cu^2+^, with one unpaired spin α electron for **3Da,** which is consistent with our findings as presented in Fig. 4.

**Fig. 5:**
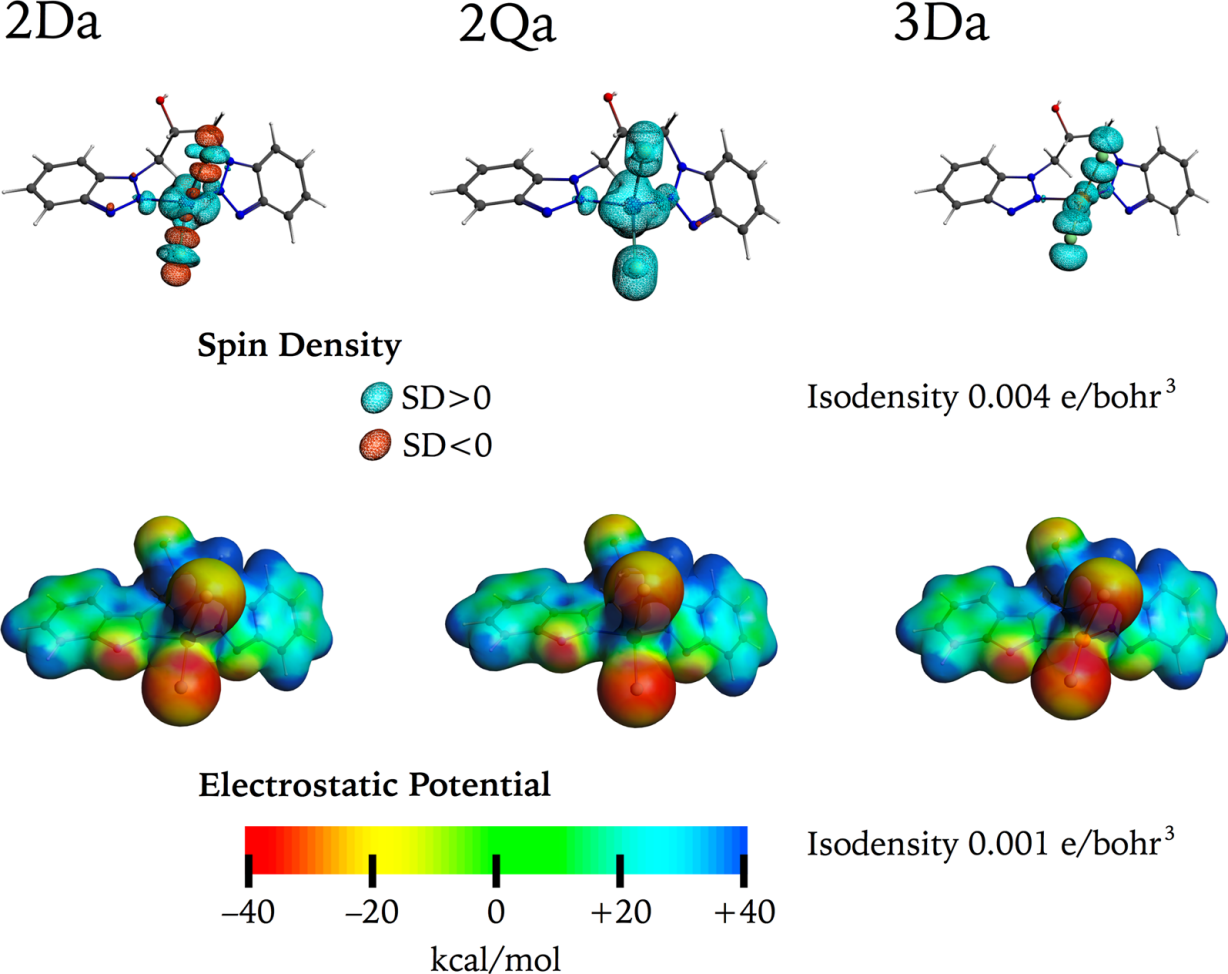
Top: Spin density for **2 Da**, **2Qa**, and **3 Da** at the isosurface cutoff value of 0.004 e/bohr^3^. Bottom: isosurfaces, corresponding to the total electron density using an isosurface cutoff value of 0.001 e/bohr^3^ mapped with the electrostatic potential between a range of − 40 and + 40 kcal/mol

The spin density of the studied complexes was calculated as the difference between the densities of electrons with spin α and those of spin β, [78] and visualized as double colored isosurfaces as presented in Fig. 5. This demonstrates that the unpaired electrons are localized at the metal centers. Despite of the differences on the electronic configuration between the cobalt and copper complexes, the electrostatic potential map, shows a similar distribution of the electron density, mainly because cobalt and copper in these complexes possess the same +2 charge (see Fig. 5). Into this context, the Hirshfield and Bader charges where calculated, as presented in Table 6. It must be noted that some differences are encountered when comparing both methodologies, the differences comparing the charges of Cl and N atoms directly linked to the metal atoms do not exceed 0.1.

**Table 6:** Hirshfield and Bader charges for **2** and **3**

Atom*	2Da		2Qa		3Da	
	Hirshfield	Bader	Hirshfield	Bader	Hirshfield	Bader
M	0.104	0.996	0.153	1.082	0.325	0.876
Cl	− 0.233	− 0.528	− 0.265	− 0.583	− 0.295	− 0.515
Cl	− 0.229	− 0.497	− 0.266	− 0.550	− 0.288	− 0.44
N	0.018	− 0.191	0.017	− 0.209	− 0.025	− 0.139
N	0.020	− 0.199	0.017	− 0.184	− 0.015	− 0.151

*Up and down references are referred to the structures presented in Figs. 4 and 5

The results obtained from the energy decomposition analysis (EDA) are shown in Table 7, for all the conformers of **2** and **3**. The [MLCl_2_] complexes (M = Co(II), Cu(II) and L = **1**) were separated into {MCl_2_} and {L} fragments, whose interaction gives the interaction energy (${\Delta E}_{inter}$) for these fragments. The ${\Delta E}_{inter}$ is decomposed into three main terms: Pauli’s repulsion (${\Delta E}_{Pauli}$), electrostatic interaction (${\Delta E}_{elstat}$) and orbital interactions (${\Delta E}_{orb}$), the last two can be grouped due to their stabilizing character, determining the ionic and covalent character of the interaction [79].

**Table 7:** Energy Decomposition Analysis (EDA) of the {MCl_2_}-{L} interaction for the different conformers of **2** and **3**. Values in kJ·mol^−1^

Complex/Multiplicity/Conformer		$\Delta {E}_{inter}$	$\Delta {E}_{Pauli}$	$\Delta {E}_{elstat}$^†^	$\Delta {E}_{orb}$^†^
**2**	Da	− 1513.54	688.25	− 440.36 (20.3%)	− 1733.92 (79.7%)
	Db	− 573.49	565.24	− 406.01 (36.5%)	− 707.27 (63.5%)
	Dc	− 384.33	1190.07	− 757.67 (49.2%)	− 783.27 (50.8%)
	Dd	− 466.85	1022.25	− 782.93 (53.9%)	− 669.57 (46.1%)
	Qa	− 650.34	545.76	− 480.03 (41.0%)	− 689.43 (59.0%)
	Qb	− 404.45	739.62	− 516.56 (46.2%)	− 602.05 (53.8%)
	Qc	− 932.62	699.69	− 470.15 (29.3%)	− 1136.50 (70.7%)
	Qd	− 546.11	564.53	− 467.82 (43.1%)	− 617.38 (56.9%)
**3**	Da	− 179.88	360.29	− 321.95 (61.6%)	− 200.72 (38.4%)
	Db	− 184.29	327.88	− 297.86 (61.1%)	− 189.76 (38.9%)
	Dc	− 167.52	619.87	− 454.44 (60.3%)	− 299.02 (39.7%)
	Dd	− 184.97	596.00	− 448.37 (59.9%)	− 300.23 (40.1%)

^†^Values in parentheses give the percentage contribution to the total attractive interactions ($\Delta {E}_{Electrostatic}+\Delta {E}_{Orbital}$)

For **3**, the stabilizing character of all the conformers is mainly due to electrostatic interactions (over 60%), while the orbital interactions only represent 40% of the contribution, with similar interaction energies. **3Da** and **3Db** show that Pauli’s repulsion overcompensates the electrostatic interactions; however, **3Dc** and **3Dd** need additional compensation from orbital interactions. The differences encountered are related to the variations in the spatial distribution of ligand **1** and the imposed steric hindrance. Whereas **3Da** and **3Db** present a distorted tetrahedral geometry, **3Dc** and **3Dd** present a distorted square planar geometry, which has a major repulsion considering the parallel interaction between the two fragments.

In the case of complex **2**, the conformers **2Da**, **2Db**, **2Dc** and **2Dd** show a similar behavior than the conformers of **3**, where the conformers with distorted tetrahedral geometry (**2Da** and **2Db**) generate a lower Pauli’s repulsion compared with those of distorted square planar geometry (**2Dc** and **2Dd**). For **2Da** and **2Db**, the major stabilizing contribution corresponds to $\Delta {E}_{orb}$ with a 79.7 and 63.5%, respectively. However, the covalent and ionic nature of the interaction are equivalent (around 50%) for **2Dc** and **2Dd** (see Table 7).

With respect to Pauli’s repulsion term, huge differences were found. **2Da** and **2Db,** the orbital interaction term produces a compensation of the $\Delta {E}_{Pauli}$ repulsive term. This is related to the orbital relaxation and the orbital mixing between the fragments which is favored by the angles at which the interaction occurs. Additionally, the larger Pauli’s repulsion contribution for **2Dc** and **2Dd** is overcompensated with both stabilizing interactions $\Delta {E}_{elstat}$ and $\Delta {E}_{orb}$.

On the other hand, the orbital interactions are the main stabilizing factor of the {MCl_2_}-{L} interaction in **2Qa**, **2Qb**, **2Qc,** and **2Qd**. All these conformers present the same distorted tetrahedral geometry, as well as **2Da** and **2Db**.

**Fig. 6:**
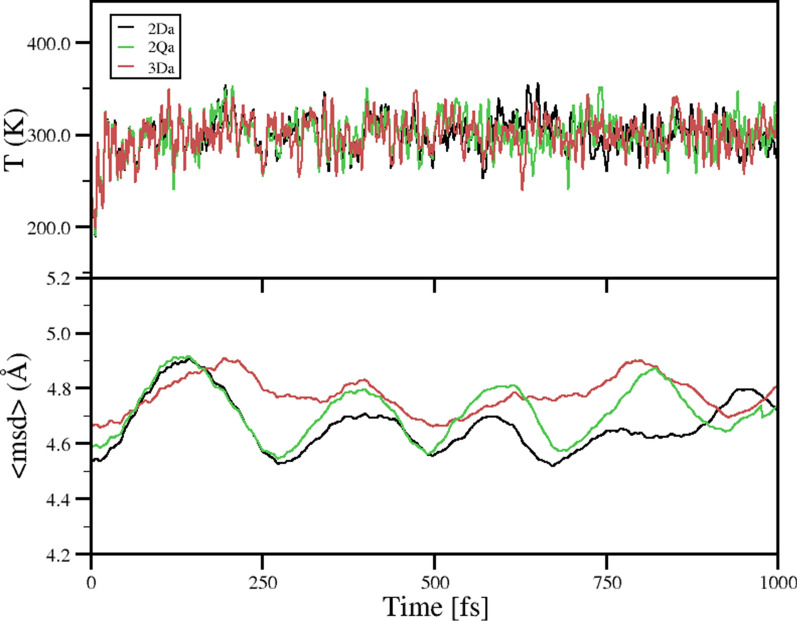
Temperature (T) and mean square displacement〈msd〉during molecular dynamics simulation for **2Da**, **2Qa**, and **3Da** complexes at 300 K, respectively

Moreover, the MD simulation results show that at 300 K, the **2** complex in its doublet and quartet state (**2Da** and **2Qa**) behave as rigid system, in which the **2** complexes show their breathing mode during simulation, which is more pronounced for **2Qa** (see Fig. 6). For **3Da**, a larger variation in the < msd > is shown, compared to the **2Da** and **2Qa** configurations. However, these variations do not exceed 0.3 Å along the complete simulation, confirming that do there is no change in the coordination modes of the complexes. Thus, according to the EDA analyzes, it is confirmed that the **2Da** is more rigid due to its larger covalent character.

In summary, considering the total bonding energy interaction and its decomposition into three main terms, **2Da** is the most favored conformer, with a large contribution from the orbital interactions and a distorted tetrahedral geometry that favored the formation of a bond between the metal center and the ligand (**1**) with main covalent character. In the case of **3**, all conformers present similar interaction energy, where the main stabilization contribution possesses an ionic character. Additionally, the MD simulations confirm that there exists a fluxional behavior that does not allow us to identify the favored conformer but confirms that the structure proposed is stable during the simulation time.

## Conclusions

In conclusion, one ligand derived from benzotriazole was synthesized employing a different methodology to that reported in the literature. Furthermore, four new complexes of Co(II) and Cu(II) were obtained from this ligand, which was characterized by spectroscopic, elemental, and thermogravimetric techniques. All the complexes (**2**–**5**) had 1:1 (*M*:*L*) stoichiometries based on characterization data, where **2** and **3** had tetrahedral geometry, while **4** and **5** had octahedral geometry. DFT calculations were carried out to propose the probable structures for **2** and **3**, where conformer **2Da** was selected as the possible geometry, while conformers of 3 had a fluxional behavior that does not allow a clear possible geometry. The findings highlight the promising role of Co(II) complexes containing 1,3-bis(benzotriazol-1-yl)-propan-2-ol as antifungal agents capable of reducing the dimorphic change in *C. albicans* and biofilm of non-*albicans* species sensitive and resistant to fluconazole. In addition, combination therapies can be examined in the future to improve selectivity in mammalian cells because synergistic interactions with caspofungin were also observed.

## Materials and methods

### General information

The metallic salts CoCl_2_·6H_2_O (purity, 98%), CuCl_2_·2H_2_O (purity, 99%), Co(CH_3_COO)_2_·4H_2_O (purity, 98%) and Cu(CH_3_COO)_2_·H_2_O (purity, 98%) were used as received from Alfa Aesar. The compounds 1H-benzotriazole (purity, 99%), 1,3-dichloro-propan-2-ol (purity, 98%), and tetrabutylammonium bromide (purity, 98%) were purchased from Sigma-Aldrich and were used as received.

Elemental analysis (C, H, and N) was performed with a Thermo Scientific™ FLASH 2000 CHNS/O Analyzer. Fourier transform infrared (FTIR) spectra were recorded on a Thermo Nicolet NEXUS FTIR spectrophotometer using ATR module. Melting points were determined on a Mel-Temp® 1101D apparatus in open capillary tubes and are uncorrected. Ultraviolet/visible (UV/vis) spectra were recorded on an Agilent Technologies Cary 100 spectrophotometer in DMSO from 200 to 800 nm in a quartz cuvette with a path length of 1 cm. Raman spectroscopy was performed in a RIBA Yovin-Ivon spectrometer using a laser with a wavelength of 786 nm. Thermogravimetric (TG) analyses of the complexes were conducted on a NETZSCH STA 409 PC/PG by evaluating 8–10 mg samples of the complexes in a nitrogen atmosphere. Samples were subjected to dynamic heating over a temperature range of 30–700 °C at a heating rate of 10 °C min^−1^. TG curves were analyzed to obtain the percent mass losses as a function of temperature. Nuclear magnetic resonance (NMR) spectra were recorded on a Bruker AscendTM-400 spectrometer at 295 K. Chemical shifts are reported in ppm relative to SiMe_4_ (^1^H) as an internal standard. ^1^H and ^13^C NMR chemical shifts (δ) are reported in parts per million (ppm) relative to TMS, with the residual solvent peak used as an internal reference; CDCl_3_ (^1^H NMR δ: 7.26 and ^13^C NMR δ: 77.2) and DMSO-*d*_6_ (^1^H NMR δ: 2.50 and ^13^C NMR δ: 39.5). High-resolution mass spectrometry (HRMS) data was obtained on an Agilent Technologies Q-TOF 6520 spectrometer via electrospray ionization (ESI) in positive ion mode**.**

### Synthesis of 1,3-bis(benzotriazol-1-yl)-propan-2-ol (1)

**Fig. 7:**
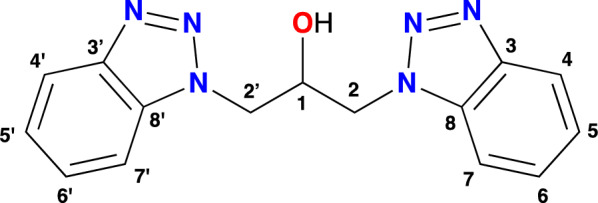
Atom numbering for signal assignment of the ^1^H and ^13^C NMR for **1**

In a Schlenk tube equipped with a reflux condenser, 1H-benzotriazole (2.500 g; 20.99 mmol), potassium hydroxide (1.211 g; 21.58 mmol), tetrabutylammonium bromide (0.997 g; 3.09 mmol), and water (20 mL) were stirred at 55 ºC for 45 min. Then, 1,3-dichloro-propan-2-ol (1.0 mL; 1.390 g; 10.78 mmol) and toluene (40 mL) were added, and the mixture was heated for 48 h at 85ºC. A white solid was generated at the interface; therefore, the reaction mixture was allowed to cool at room temperature (rt) and filtered under vacuum. The filtrate was extracted with 4 portions of water (20 mL), separated and dried with sodium sulfate. The solution was concentrated to dryness under vacuum to give a yellow solid. Both solids were mixed and purified by recrystallization with tetrahydrofuran:pentane and the ligand was obtained as a white solid (structure of the ligand **1** is shown in Fig. 7 with the respective atom numbering).

Yield 2.225 g (72.03%). M.p.: 185–187 °C. FTIR (ATR, cm^−1^): 3368 (OH), 3067 (C-H), 2924 (C-H), 1616, 1589, 1497, 1454 (N–N), 1420, 1358, 1304, 1281, 1265, 1227 (C-N), 1169, 1134, 1092 (C-O), 1038, 1011, 922, 872, 849, 799, 779, 768, 737, 667, 625, 606, 575, 548, 517, 471, 428. Raman (cm^−1^): 3369 (O–H), 3314, 3168, 3071, 2991, 2950, 2918, 1762, 1590 (benzene ring), 1498 (benzene ring), 1379 (triazole ring), 1270, 1233 (triazole ring), 1165 (N–N-N, triazole ring), 1122, 1003, 879, 768, 620 (triazole ring), 542 (triazole ring), 331, 181.

^1^H NMR (400.1 MHz, DMSO-d_6_) δ 8.04 (d, 2H, 4, 4’), 7.91 (d, 2H, 7, 7’), 7.56 (t, 2H, 6, 6’), 7.40 (t, 2H, 5, 5’), 5.62 (d, 1H, OH), 4.99 (dd, 2H, 2, 2’), 4.81 (dd, 2H, 2, 2’), 4.56 (s, 1H, 1). ^13^C NMR (DMSO-d_6_, 101 MHz) δ 145.15 (C3, C3’), 133.78 (C8, C8’), 127.17 (C6, C6’), 123.89 (C5, C5’), 119.04 (C4, C4’), 111.26 (C7, C7’), 68.90 (C1), 51.41(C2, C2’). MS–ESI (m/z, ES +) calcd. For [M + H]^+^: 295, found: 295. UV/Vis bands λ_max_, nm (ε, L mol^−1^ cm^−1^): 204 (76,272), 262 (23,597), 280 (16,771). Anal. Calcd. for C_15_H_14_N_6_O: C 61.21; H 4.79; N 28.55. Found: C 61.25; H 4.84; N 28.49%.

### Synthesis of the complexes

**Fig. 8:**
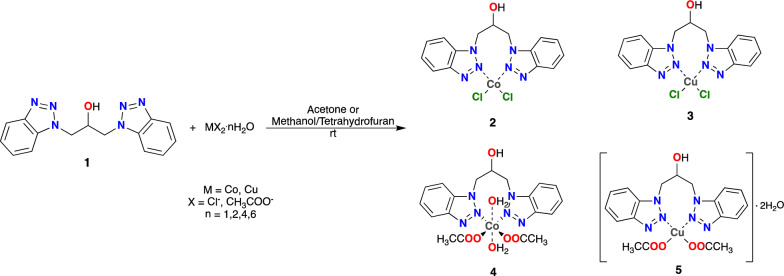
General procedure of synthesis of complexes (**2**–**5**)

The general procedure for the synthesis of complexes (**2**–**5**) is shown in Fig. 8.

#### Synthesis of [Co{1,3-bis(benzotriazol-1-yl)-propan-2-ol-N,N}Cl_2_] (2)

(**1**) (0.35 mmol; 103.4 mg) was dissolved in acetone (17 mL), and CoCl_2_·6H_2_O (0.34 mmol; 80.9 mg) in acetone (5 mL) was added to this mixture. The resulting solution was stirred for 3 h at rt. This mixture was centrifuged at 400 rpm for 9 min, washing with acetone and ethyl ether removing the liquid phase between each wash. Then, the solvent was evaporated to dryness to give a blue solid.

Yield: 132.2 mg (91.7%). M.p.: 341–346 °C (decomposition). FTIR (ATR, cm^−1^): 3491 (O–H), 3098 (C-H), 2928 (C-H), 1709, 1597, 1497, 1458 (N–N), 1435, 1393, 1373, 1350, 1312, 1288, 1231 (C-N), 1184, 1169, 1146, 1026 (C-O), 949, 876, 853, 806, 779, 756, 741, 671, 621, 579, 540, 521, 428. Raman (cm^−1^): 3074, 2968, 2933, 2920, 1599 (benzene ring), 1494 (benzene ring), 1388 (triazole ring), 1364, 1293, 1226 (triazole ring), 1126, 1008, 951 (M-L), 875, 775, 664, 620 (triazole ring), 571, 539 (triazole ring), 497, 466, 343 (M-Cl), 304, 193. UV/Vis bands λ_max_, nm (ε, L mol^−1^ cm^−1^): 204 (39,461), 263 (12,404), 280 (9304), 530 (25). Anal. Calcd. for C_15_H_14_Cl_2_CoN_6_O: C 42.48; H 3.33; N 19.81. Found: C 42.51; H 3.39; N 19.78%.

#### Synthesis of [Cu{1,3-bis(benzotriazol-1-yl)-propan-2-ol-N,N}Cl_2_] (3)

(**1**) (0.34 mmol; 99.6 mg) was dissolved in acetone (14 mL), and CuCl_2_·2H_2_O (0.33 mmol; 55.9 mg) in acetone (8 mL) was added to this mixture. The resulting solution was stirred for 3 h at rt. This mixture was centrifuged at 400 rpm for 9 min, washing with acetone and ethyl ether removing the liquid phase between each wash. Then, the solvent was evaporated to dryness to give a pea-green solid.

Yield: 95.8 mg (67.7%). M.p.: 195–199 °C (decomposition). FTIR (ATR, cm^−1^): 3379 (O–H), 1593, 1493, 1458 (N–N), 1323, 1288, 1234 (C-N), 1165, 1092 (C-O), 1003, 945, 872, 779, 745, 667, 652, 575, 513, 432. Raman (cm^−1^): 3448 (O–H), 3329, 3293, 3172, 3070, 2946, 1751, 1589 (benzene ring), 1490 (benzene ring), 1456, 1373 (triazole ring), 1281, 1233 (triazole ring), 1170 (N–N-N, triazole ring), 1126, 997, 937 (M-L), 876, 777, 619 (triazole ring), 544 (triazole ring), 375 (M-Cl), 265. UV/Vis bands λ_max_, nm (ε, L mol^−1^ cm^−1^): 203 (60,623), 262 (17,372), 276 (11,714), 860 (106). Anal. Calcd. for C_15_H_14_Cl_2_CuN_6_O: C 42.02; H 3.29; N 19.60. Found: C 42.05; H 3.31; N 19.55%.

#### Synthesis of [Co{1,3-bis(benzotriazol-1-yl)-propan-2-ol-N,N}(H_2_O)_2_(CH_3_COO)_2_] (4)

(**1**) (0.34 mmol; 100.3 mg) was dissolved in tetrahydrofuran:methanol (4:1, 15 mL), and Co(CH_3_COO)_2_·4H_2_O (0.34 mmol; 83.7 mg) in tetrahydrofuran:methanol (3:2, 5 mL) was added to this mixture. A color change of the solution to orange was immediately observed. The resulting solution was stirred for 2 h at rt. The solution was concentrated to dryness under a vacuum to give a purple solid, which was washed with acetone and ethyl ether, removing the liquid phase between each wash. Then, the solvent was evaporated to dryness to give a purple solid.

Yield: 131.1 mg (76.0%). M.p.: 162–166 °C (decomposition). FTIR (ATR, cm^−1^): 3367 (O–H), 3067 (C-H), 2924 (C-H), 1558 (C = O), 1497, 1416 (N–N), 1342, 1304, 1227 (C-N), 1165, 1134, 1096 (C-O), 1015, 872, 779, 741, 667, 613, 548, 513, 471, 432. Raman (cm^−1^): 3367 (O–H), 3062, 2989, 2947, 2917, 1586 (benzene ring), 1488 (benzene ring), 1451, 1385 (triazole ring), 1308, 1267, 1227 (triazole ring), 1166 (N–N-N, triazole ring), 1109, 998, 930 (M-L), 878, 772, 615 (triazole ring), 179, 74 (M–O_(acetate)_). UV/Vis bands λ_max_, nm (ε, L mol^−1^ cm^−1^): 203 (32,949), 261 (10,264), 280 (8426), 514 (28). Anal. Calcd. for C_19_H_24_CoN_6_O_7_: C 44.98; H 4.77; N 16.56. Found: C 44.99; H 4.80; N 16.53%.

#### Synthesis of [Cu{1,3-bis(benzotriazol-1-yl)-propan-2-ol-N,N}(CH_3_COO)_2_]⋅2H_2_O (5)

(**1**) (0.34 mmol; 100.5 mg) was dissolved in tetrahydrofuran:methanol (4:1, 15 mL), and Cu(CH_3_COO)_2_·H_2_O (0.33 mmol; 66.8 mg) in tetrahydrofuran:methanol (3:2, 5 mL) was added to this mixture. The resulting solution was stirred for 5 h at reflux. The reaction mixture was allowed to cool at rt and concentrated to dryness under vacuum to give a green solid, which was washed with acetone and ethyl ether removing the liquid phase between each wash. Then, the solvent was evaporated to dryness to give a dark green solid.

Yield: 156.1 mg (93.1%). M.p.: 199–203 °C (decomposition). FTIR (ATR, cm^−1^): 3368 (O-H), 3067 (C-H), 2920 (C-H), 1609 (C = O), 1497, 1423 (N-N), 1300, 1281, 1227 (C-N), 1165, 1134, 1084 (C-O), 1003, 934, 899, 872, 779, 745, 683, 625, 548, 513, 432. Raman (cm^−1^): 3335 (O-H), 3173, 3066, 2928, 1590 (benzene ring), 1532, 1495 (benzene ring), 1447, 1379 (triazole ring), 1287, 1267, 1229 (triazole ring), 1165 (N-N-N, triazole ring), 1122, 1003, 938 (M-L), 884, 838, 775, 698, 625 (triazole ring), 542 (triazole ring), 511, 302, 220, 186, 116 (M–O_(acetate)_). UV/Vis bands λ_max_, nm (ε, L mol^−1^ cm^−1^): 204 (49,112), 262 (13,205), 278 (9498), 424 (192), 450 (262), 714 (38). Anal. Calcd. for C_19_H_24_CuN_6_O_7_: C 44.57; H 4.72; N 16.41. Found: C 44.62; H 4.81; N 16.31%.

### Biological studies

#### Microorganisms and mammalian cells

The study was carried out on eight strains of *Candida *spp. Four reference strains obtained from the American Type Culture Collection-ATCC (*C. albicans* 90,028; *C. tropicalis* 66,029; *C. glabrata* MYA2950; *C. parapsilosis* 22,019) and clinical isolates resistant to fluconazole donated and characterized genotypically and phenotypically by Corporación para Investigaciones Biológicas—CIB, Medellín, Colombia (*C. albicans* CAPF-13; *C. tropicalis* CAPF-01; *C. glabrata* CAPF-07; *C. parapsilosis* 24,754). All yeasts were cultured on saboraud agar (OXOID Ltd., Basingstoke, Hampshire, UK) at 35 °C. Fresh cultures were used for each experiment.

Macrophage J774.A1 (ATCC® TIB-67™) was donated by Cellular and Functional Biology and Biomolecular Engineering Group from the Universidad Antonio Nariño, Colombia. The cells were cultured in Dulbecco's modified Eagle's medium (DMEM) (Gibco, USA), supplemented with 10% inactivated fetal bovine serum (Gibco, USA), 1% penicillin–streptomycin (Gibco, USA) and kept under conditions of 95% humidity, 5% CO_2_ and 37 °C.

#### Susceptibility on planktonic cells of *Candida sp*

The minimum inhibitory concentrations (MICs) for strains of *Candida* spp were determined by the microdilution method according to Clinical & Laboratory Standards Institute (CLSI) guidelines, protocol M27-A4. Fluconazole (FCZ) and itraconazole (ITZ) obtained from Sigma-aldrich were used as reference drugs. The minimum fungicidal concentration (CFM) was determined from subcultures on saboraud agar of the MIC and concentrations above the MIC. The CFM was the concentration of the compound in which the growth of ≤ 3 CFU was observed after 24 h of incubation at 35 ºC.

#### In vitro drug interaction assay

The modified fixed-ratio isobologram method described by Quinton L. Fivelman *et* *al.* was used [80]. Pharmacological interactions between drug A and drug B were prepared from MIC. Concentrations equal to 8X MIC, 4X MIC, 2X MIC, MIC, 1/2 MIC, and 1/4 MIC were prepared and combined inversely (Additional file 1: Table S3).

U-bottom plates were inoculated with 0.5 × 10^2^ cells/mL—2.5 × 10^3^ cells/mL of *Candida *spp. incubated for 24 h, 37 °C. Fractional MICs were obtained visually as the concentration that inhibits 50% of the initial inoculum. Fractional inhibitory concentrations (FIC) are calculated from the MIC obtained using Eq. (1).

1 $$FIC \left(drug\right)=\frac{MIC (drug\,in\,combination)}{MIC (drug\,alone)}$$

The ∑FIC index is obtained from the sum of the FIC of the drugs in each combination. Its value defines whether the interaction is synergistic (< 0.5), additive (0.51—0.99), indifferent (1—3.9), or antagonistic (> 4).

#### Antibiofilm activity

To evaluate the effect of cobalt(II) complexes on the resulting biofilm, 10^6^ cells/ml of *Candida* spp. were grown in RPMI 1640. 200 μL of each culture was added to 96-well flat-bottom microtiter plates and incubated for 24 h at 37 °C with shaking (50 rpm) to allow biofilm formation as was previously described [81]. Afterward, *Candida* biofilms were rinsed three times with PBS to remove planktonic cells. Different concentrations of cobalt (II) complexes were added to yield final concentrations among 1/2MIC-4XMIC. Plates were incubated without shaking for 24 h, at 37 °C. Untreated cells and RPMI 1640 without yeast were included as positive and negative controls, respectively. The metabolic activity of biofilms was determined using a semi-quantitative 2,3-bis-(2-methoxy-4-nitro-5-sulfophenyl)-2H-tetrazolium-5-carboxanilide (XTT, Cayman Chemical) reduction assay. In brief, stock solutions of XTT in PBS (0.5 g/L) and Menadione in acetone (10 mM) were prepared and stored at -80 °C. Prior to use XTT/menadione solutions were freshly prepared in a ratio 10:1. 100 μL of XTT/Menadione mixture was then added to each well. Plates were incubated for 3 h, at 37 °C, in the dark, and absorbance was measured at 490 nm. The sessile minimum inhibitory concentrations (SMIC_50_) were calculated.

#### Filamentation assay

The inhibitory effect of Co(II) complexes on the switch from yeast to hyphae of *C.l albicans* was tested, according to described by Sun *et* *al.*, 2015 [82]. Briefly, cells were grown at 37 °C on YPD broth (1% yeast extract, 2% peptone, and 2% glucose), with rotary shaking at 200 rpm, overnight. Then, cells were harvested by centrifugation and washed twice with ultrapure water. 2.5 × 10^6^ cells/mL were transferred to RPMI 1640 supplemented with 0.5% GlcNAc; 0.5% peptone, and 0.3% KH_2_PO_4_, with or without metallic complexes (control). The compounds were added in a concentration range of 7.8–62.5 µg/mL. The plates were incubated at 37 °C during 4 h. Lastly, cell morphology was recorded by counting at least 200 cells, discriminating between yeast cells and hyphae. The results were expressed as the percentage of the mycelium, and the inhibition percent was calculated. Ten repetitions were established with each concentration. The assays were repeated in two independent moments.

#### In vitro cytotoxicity assay

The in vitro effect of the complexes, ligands, and salts on the viability of J774.A1 macrophages was determined by the colorimetric method using the tetrazolium salt (3-(4,5-dimethylthiazol-2-yl)-2,5-diphenyltetrazolium bromide (MTT, Sigma-Aldrich). In summary, a cell density of 1 × 10^5^ cells/mL in monolayer was exposed for 72 h with the metal complexes in concentration ranges 300–11.1 μg/mL. Then, MTT was added to each well (10%) for 4 h, and the optical density determined at 595 nm using an iMark™ Microplate Absorbance Reader (BioRad, Madrid, Spain). The cytotoxicity percentage was calculated with the equation: [(OD450nm control–OD450nm treatment)/OD450nm treatment)] × 100. The results were expressed as Cytotoxic Concentration 50 (CC_50_) determined by sigmoidal regression using Msxlfit software (GO Business Solution, Guildford, UK) [83–85].

#### Statistical analysis

Data analysis of filamentation assays was performed with the statistical package IBM SPSS Statistics 25.0. The effect of different concentrations of Co(II) complexes on dimorphic transition of *C. albicans* were determined using the Kruskal–Wallis test. A *p* value < 0.05 was defined as statistically significant. The cytotoxic concentration 50 (CC_50_) and 90 (CC_90_) were calculated by sigmoidal regression from the percentages of inhibition using Msxlfit software (GO Business Solution, Guildford, UK). Graphs were generated using Microsoft Excel.

### Computational details

#### Density functional theory calculations

The Amsterdam Density Functional (ADF) package [86] was used to calculate geometrical, electronic structures, and optical properties at the relativistic level of theory. A spin-unrestricted scheme was employed for open shell systems. The scalar relativistic effects were incorporated into the calculations by means of a two-component Hamiltonian with the zeroth-order regular approximation (ZORA) [87, 88]. The generalized gradient approximation (GGA) with the exchange–correlation functional by Perdew-Burke-Ernzerhof (PBE) [89, 90] was used for all calculations. Additionally, the triple-ζ quality Slater-type orbital (STO) basis set with one polarization functions (TZP) and two polarization functions (TZ2P) [91] were used for non-metallic (H, C, N, O and Cl) and metallic atoms (Co and Cu), respectively. Finally, the Stefan Grimme dispersion correction functional (GRIMME3) with Becke and Johnson damping function (BJDAMP) [92–94] were included for all calculations.

According to the experience of authors specialized in DFT calculation, the most suitable methodology to address the Cobalt and Copper complexes present in this manuscript correspond to SR-PBE-D3BJ/TZP-TZ2P. In this sense, benchmark studies show that PBE including vdW corrections is a suitable functional to study inorganic complexes [95, 96]. To account for weak interactions, dispersion corrections were included in these calculations, mainly due to the different conformations that ligands can adopt, since the metal center has a positive charge which reduces the effective number of electrons for the dispersion interactions. The Perdew–Burke–Ernzerhof (PBE) density functional was chosen based in previous literature studies [97–102].

Spin Density of the studied complexes was calculated as the difference between the densities of electrons with spin α and those of spin β, and visualized as double colored isosurfaces [78]. Population analyses were carried out on the basis of the natural population analysis (NPA) scheme by using the NBO5 standalone suite [103]. Also, the Hirshfield analysis, that generates a charge value by comparing the integral of the charge density over space weighted by the relative fraction of the (initial) density of that fragment in the total initial (sum-of-fragments) density [104, 105]. Finally, a real-space partition of the electronic density based on the quantum theory of atoms in molecules (QTAIM) developed by Richard Bader was employed as implemented in ADF [106].

#### Energy decomposition analysis (EDA)

The bonding analysis focuses on the interaction energy of a bond formed between two fragments in a specific electronic state with a frozen geometry. In the case of the complexes studied here, the complexes [MCl_2_L] were fragmented in {MCl_2_} and {L} corresponding to ligand (**1**), and their electronic energy and wavefunctions were obtained by performing single-point calculations. Subsequently, the fragment’s wavefunctions were combined to obtain the molecular wavefunction and corresponding binding interaction energy [107]. The EDA scheme proposed by Morokuma-Ziegler [108–111], dissects the binding interaction energy into three main components: electrostatic interaction, Pauli’s repulsion, and orbital interaction (see Eq. (2)).

2 $${\Delta E}_{inter}={\Delta E}_{elstat}+{\Delta E}_{Pauli}+{\Delta E}_{orb}$$

The electrostatic component (${\Delta E}_{elstat}$) corresponds to the classical interaction form, the superposition of the unperturbed fragment densities at the molecular geometry, considering the effects associated with Coulombic attraction and repulsion. This component has a stabilizing character. The Pauli’s component (${\Delta E}_{Pauli}$) is associated with the principle through explicit antisymmetrization and renormalization of the product wavefunction and the energy change between the superposition of the unperturbed wavefunction of the isolated fragment and the molecular wavefunction of the conformer. The Pauli’s term has a destabilizing character. Finally, the orbital mixing component (${\Delta E}_{orb}$) has a stabilizing influence due to the mixing of occupied and unoccupied orbitals that generate a relaxation of the molecular system and can involve charge transfer and polarization effects [107].

#### Molecular dynamics (MD) simulations

In order to explore the thermodynamic stability of **2Da** and **3Da** complexes, we have carried out Molecular dynamics MD simulations as implemented in the Orca quantum chemistry package [112]. We have used the default Velocity Verlet algorithm in the NVT ensemble at the PBE/def2-SVP level of theory. A Timestep of 0.5 femtoseconds and initial velocities according to a temperature of 300 K are used. Temperature is maintained at 300 K using a Berendsen thermostat [113]. The simulations are performed for a time of 30 ps with 10 fs of time step. The behavior of the mean square displacement 〈msd〉 as a function of time allows us to determine the average bond-length variations during MD simulation. In order to analyze the temperature stability of **2Da** and **3Da** complexes, we evaluated the mean-square displacement 〈msd〉 defined in Eq. (3).

3 $$<msd>=\frac{1}{N}{\sum }_{i=1}^{N}{\left[{r}_{i}\left(t\right)-{r}_{i}\left(0\right)\right]}^{2}$$

where r_i_(t) is the position vector of the i-th atom at the time t and N is the total number of atoms in the system. The dynamical behavior of boron clusters has been rationalized by the〈msd〉parameter [114]. In a rigid system, the 〈msd〉 parameter remains constant, whereas in a non-rigid system the 〈msd〉 show variations as a function of the time.

### Supplementary Information

**Additional file 1.** Additional figures and tables.

## Data Availability

No datasets were generated or analyzed during the current study.
