# Supplementary material for: Antifungal activity of Co(II) and Cu(II) complexes containing 1,3-bis(benzotriazol-1-yl)-propan-2-ol on the growth and virulence traits of fluconazole-resistant Candida species: synthesis, DFT calculations, and biological activity
Source: BMC Chem. 2023 Oct 10;17(1):135. doi: 10.1186/s13065-023-01037-7 (PMC10563319; doi:10.1186/s13065-023-01037-7)
Supplement: Supplementary file 1 — Additional file 1. Additional figures and tables. [file 13065_2023_1037_MOESM1_ESM.pdf]

Supplementary Material

Antifungal activity of Co(II) and Cu(II) complexes containing 1,3-bis(benzotriazol-1-yl)-propan-2-ol on the growth and virulence traits of fluconazole-resistant *Candida* species: Synthesis, DFT calculations, and biological activity

Ricardo A. Murcia-Galan[1], Sandra M. Durán[2], Sandra M. Leal-Pinto[2], Martha V. Roa-Cordero[2], Jose D. Vargas[2], Laura V. Herrera[3], Alvaro Muñoz-Castro[4], Desmond MacLeod-Carey[5], Tonny W. Naranjo[6,7], Peter L. Rodríguez-Kessler,[8] John J. Hurtado[1*]

[1]Grupo de investigación en Química Inorgánica, Catálisis y Bioinorgánica, Departamento de Química, Universidad de los Andes, Carrera 1 No. 18A-12, 111711, Bogotá-Colombia; ra.murcia@uniandes.edu.co; jj.hurtado@uniandes.edu.co
[2]Universidad de Santander, Facultad de Ciencias Médicas y de la Salud, Calle 70 No. 55-210, Bucaramanga-Colombia; sandramduranbarajas@gmail.com sa.leal@mail.udes.edu.co; ma.roa@mail.udes.edu.co; vargasjosedavid.7.3@gmail.com
[3]Grupo Sistema Estomatognático y Morfofisiología (SEMF). Departamento de Ciencias Básicas, Universidad Santo Tomás seccional Bucaramanga, cra 27 No. 180-395, Bucaramanga-Colombia; laura.herrera01@ustabuca.edu.co
[4]Facultad de Ingeniería, Arquitectura y Diseño, Universidad San Sebastián, Bellavista 7, Santiago, 8420524 Chile; alvaro.munozc@uss.cl
[5]Universidad Autónoma de Chile, Facultad de Ingeniería, Instituto de Ciencias Químicas Aplicadas, Inorganic Chemistry and Molecular Materials Center, El Llano Subercaseaux 2801, Santiago, Chile; desmond.macleod@uautonoma.cl
[6] Corporación para Investigaciones Biológicas (CIB), Experimental and Medical Micology Group, Medellin 050010, Colombia; tnaranjo@cib.org.co
[7] Universidad Pontificia Bolivariana, Facultad de Medicina, Medellín 050034,Colombia; tnaranjo@cib.org.co
[8]Centro de Investigaciones en Óptica A.C., Loma del Bosque 115, Col. Lomas del Campestre, León, Guanajuato, 37150, México; plkessler@cio.mx
* Correspondence: jj.hurtado@uniandes.edu.co; Tel.: +57-1-3394949 (ext. 3468), ORCID: https://orcid.org/0000-0002-0511-9719

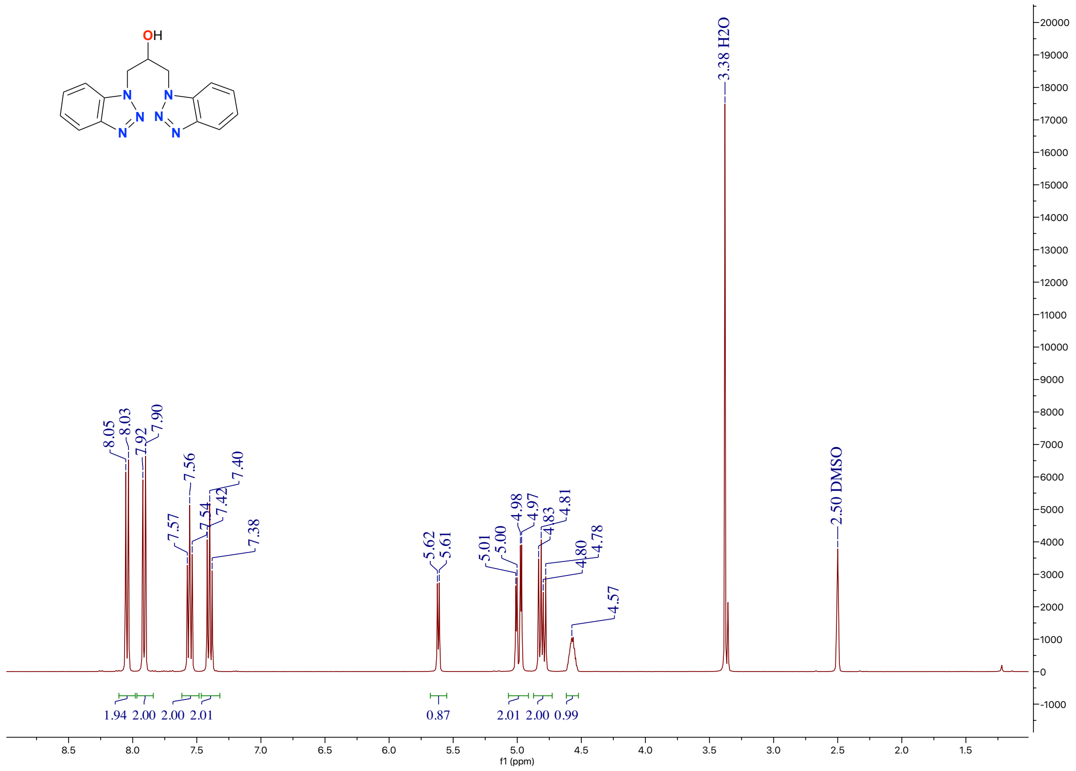

Figure S1. ^{1}H NMR spectrum of 1,3-bis(benzotriazole-1-yl)propan-2-ol (**1**) in DMSO-d_6

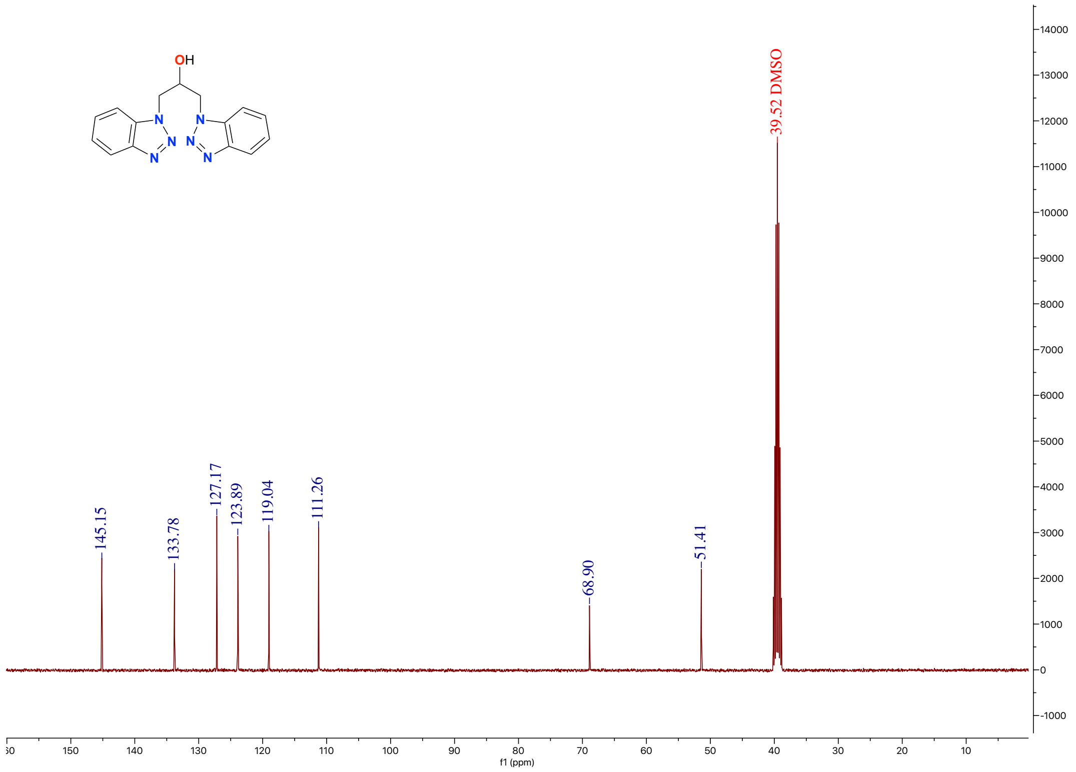

Figure S2. ^{13}C NMR spectrum of 1,3-bis(benzotriazole-1-yl)propan-2-ol (**1**) in DMSO-d_6

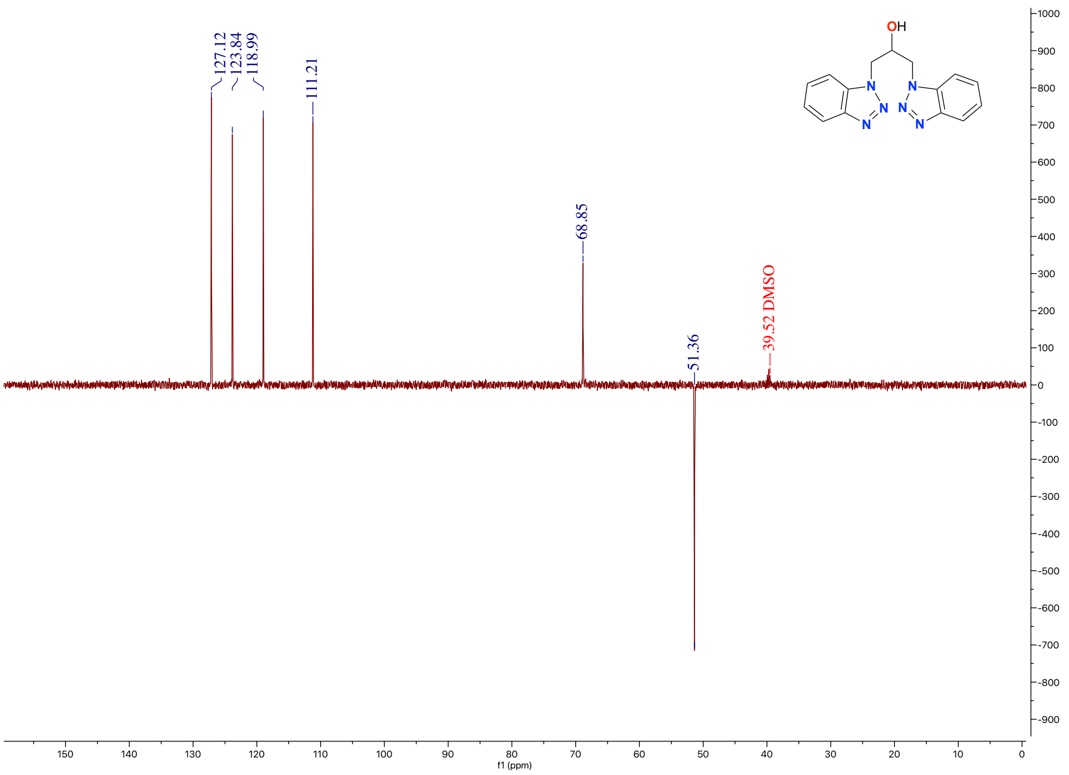

Figure S3. DEPT NMR spectrum of 1,3-bis(benzotriazole-1-yl)propan-2-ol (**1**) in DMSO-d_6

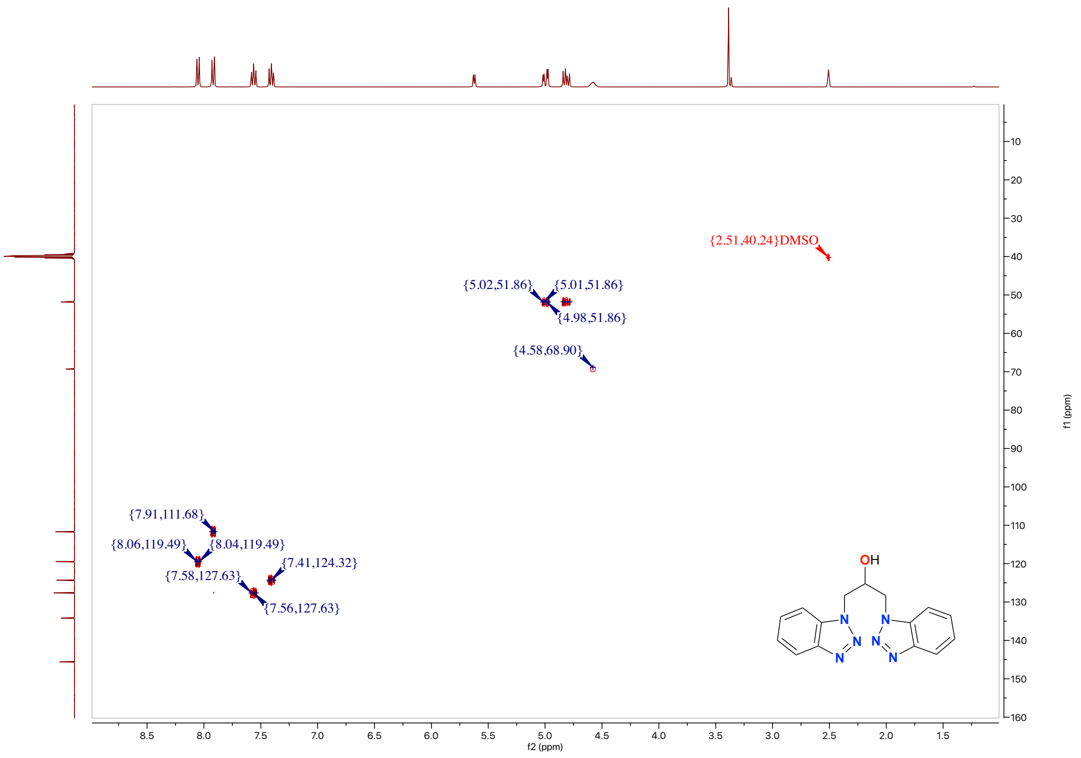

Figure S4. HSQC NMR spectrum of 1,3-bis(benzotriazole-1-yl)propan-2-ol (**1**) in DMSO-d_6

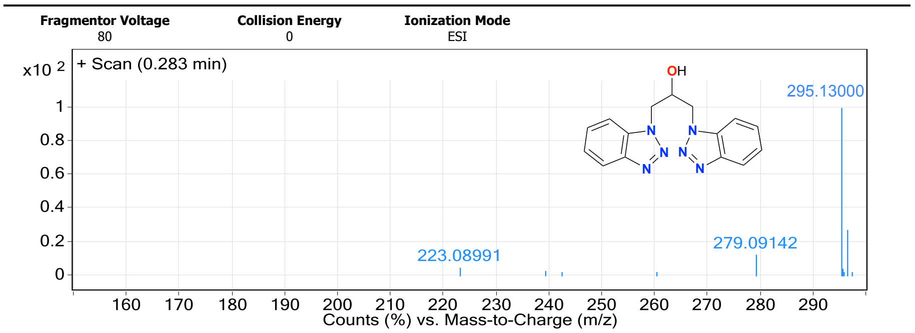

Figure S5. MS spectrum of 1,3-bis(benzotriazole-1-yl)propan-2-ol (**1**)

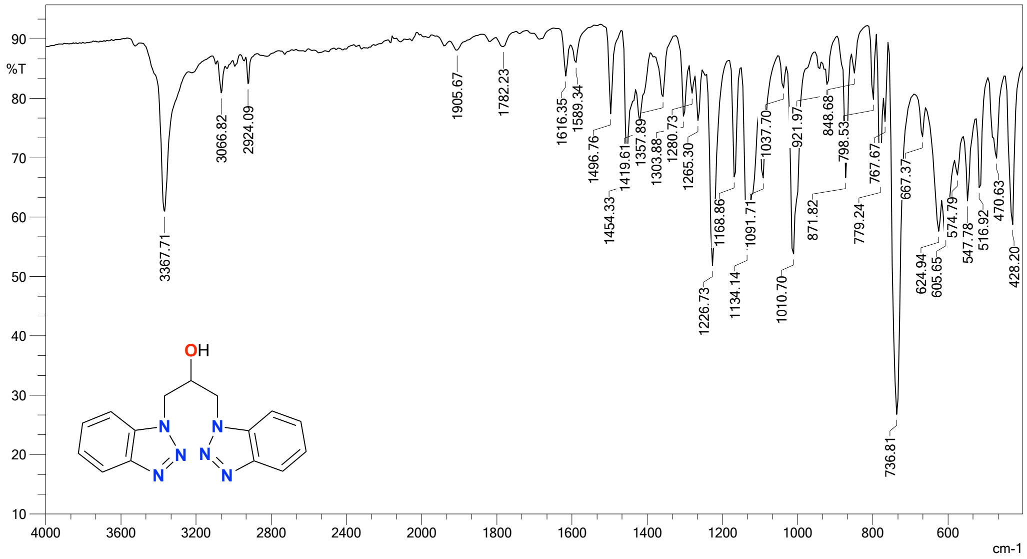

Figure S6. FT-IR (ATR) spectrum of 1,3-bis(benzotriazole-1-yl)propan-2-ol (**1**)

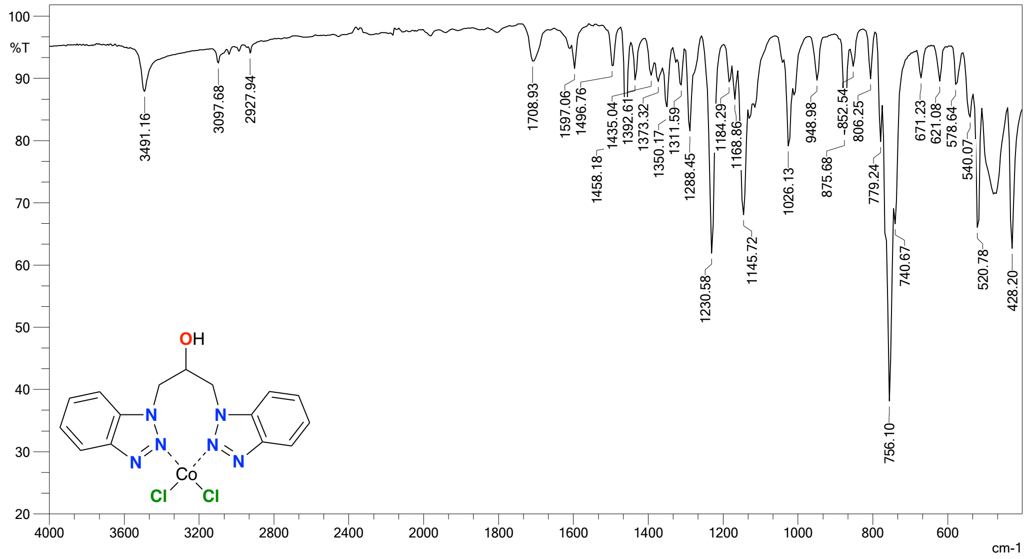

Figure S7. FT-IR (ATR) spectrum of dichloro[1,3-bis(benzotriazole-1-yl)propan-2-ol-N,N’]cobalt(II) (**2**)

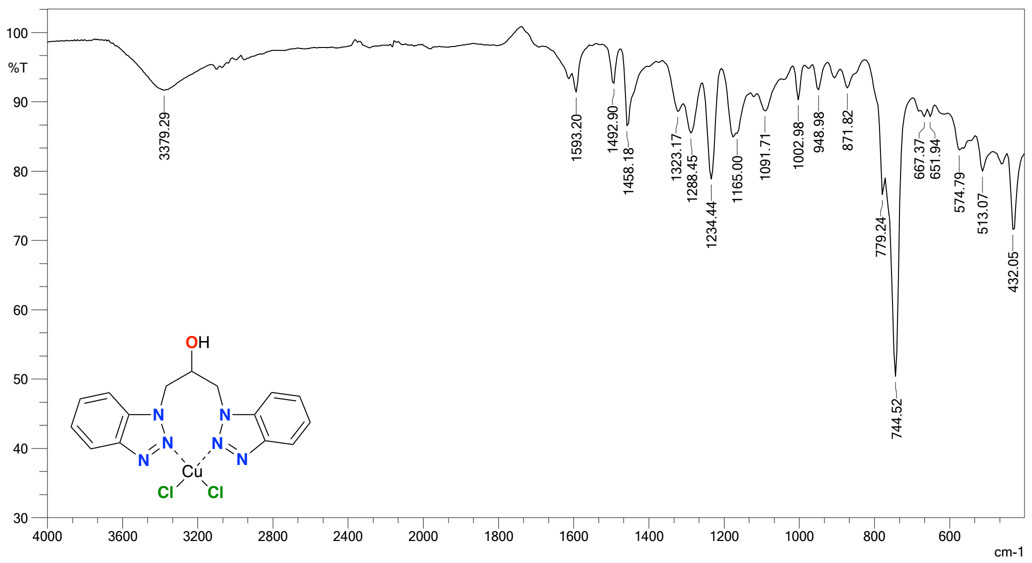

Figure S8. FT-IR (ATR) spectrum of dichloro[1,3-bis(benzotriazole-1-yl)propan-2-ol-N,N']copper(II) (**3**)

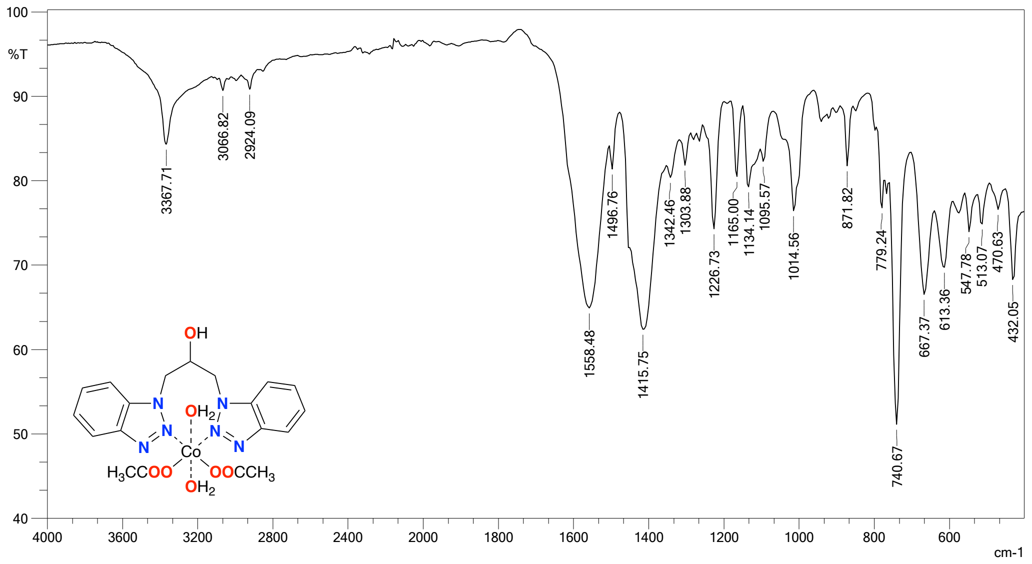

Figure S9. FT-IR (ATR) spectrum of diacetate-diaqua-[1,3-bis(benzotriazole-1-yl)propan-2-ol-N,N']cobalt(II) (**4**)

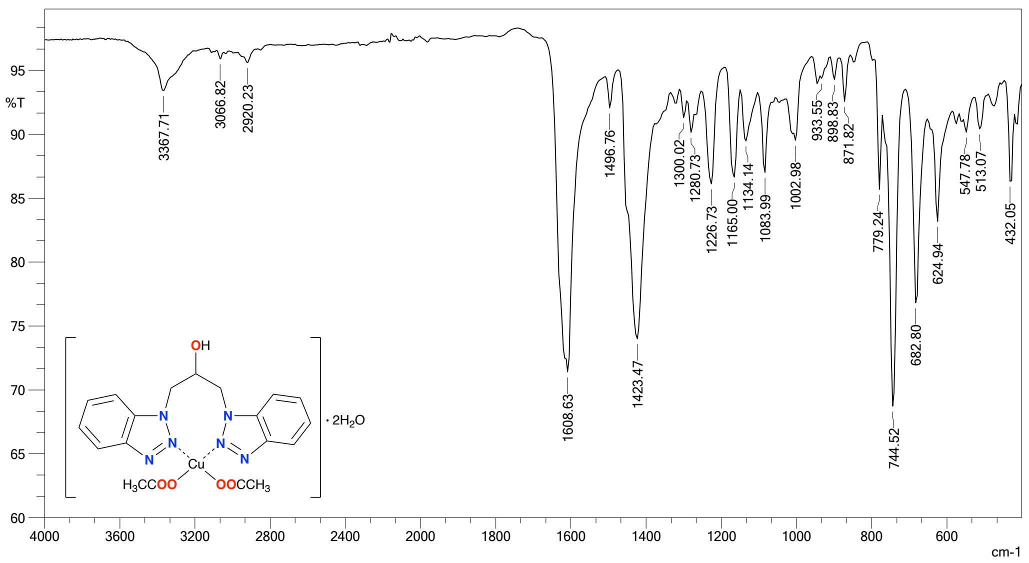

Figure S10. FT-IR (ATR) spectrum of diacetate[1,3-bis(benzotriazole-1-yl)propan-2-ol-N,N']copper(II) dihydrated (**5**)

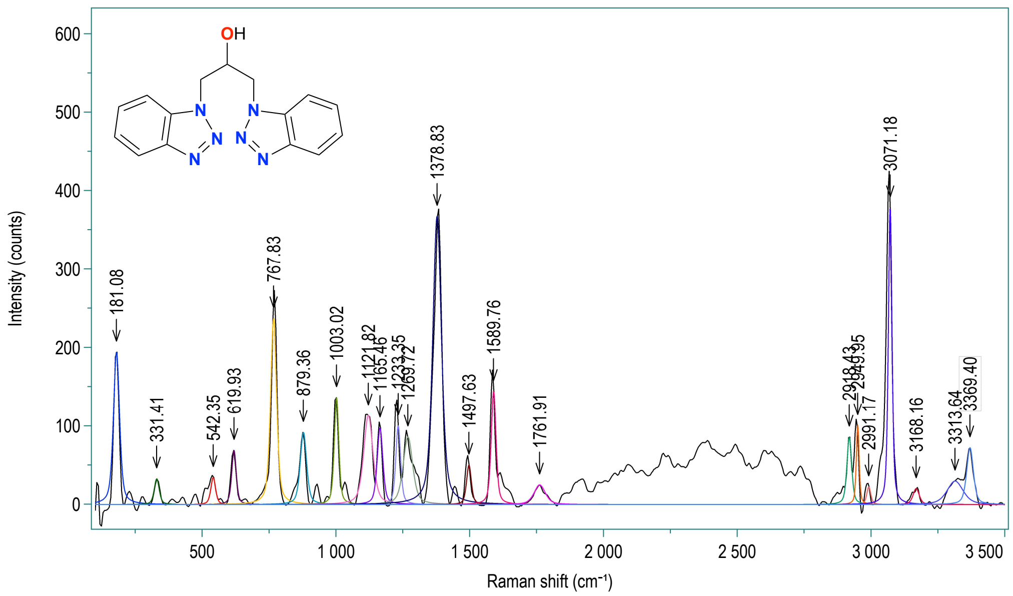

Figure S11. Raman spectrum of 1,3-bis(benzotriazole-1-yl)propan-2-ol (**1**)

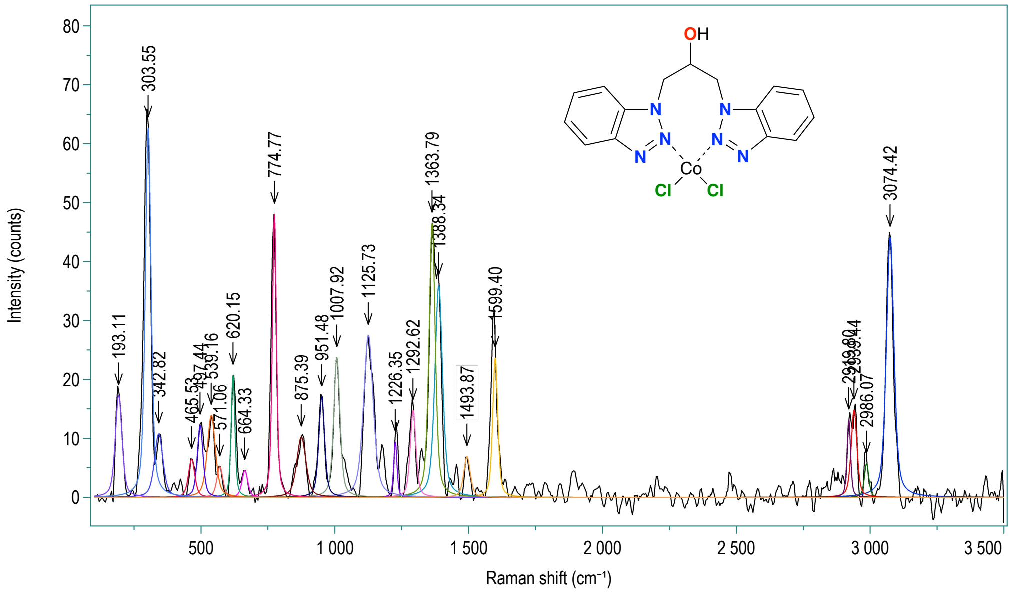

Figure S12. Raman spectrum of dichloro[1,3-bis(benzotriazole-1-yl)propan-2-ol-N,N’]cobalt(II) (**2**)

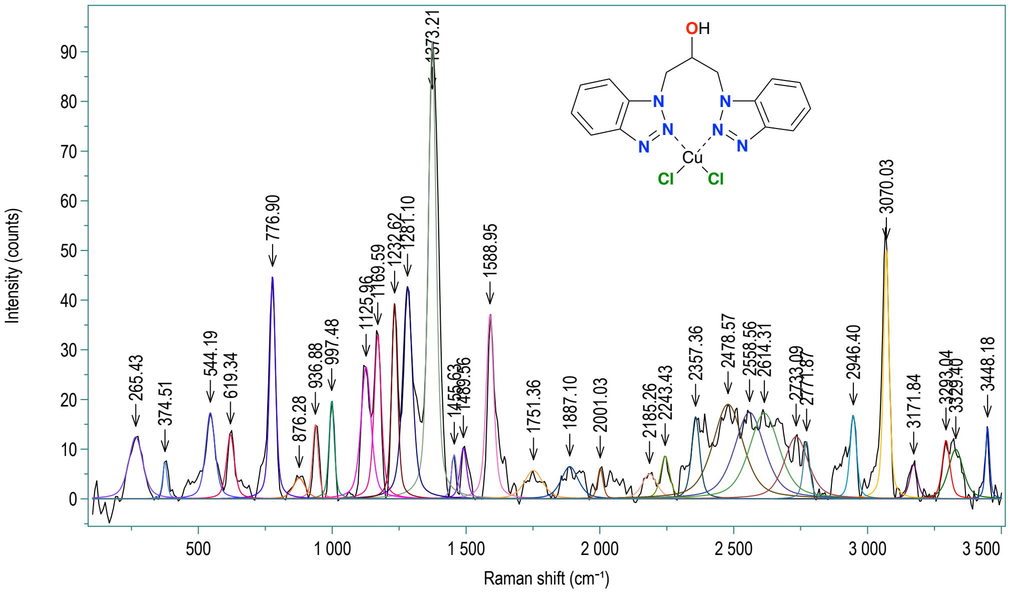

Figure S13. Raman spectrum of dichloro[1,3-bis(benzotriazole-1-yl)propan-2-ol-N,N’]copper(II) (**3**)

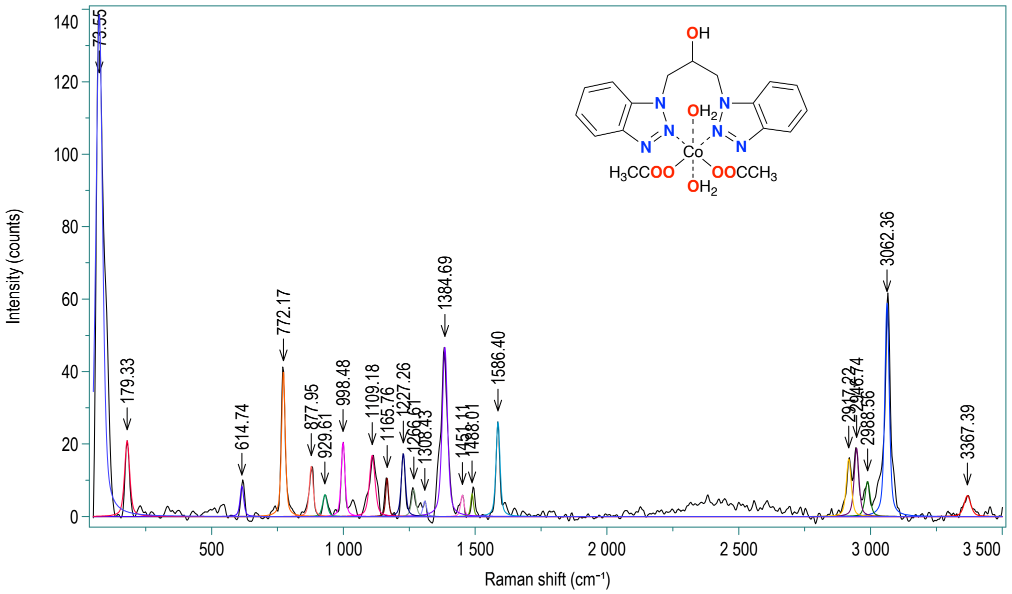

Figure S14. Raman spectrum of diacetate-diaqua-[1,3-bis(benzotriazole-1-yl)propan-2-ol-N,N']cobalt(II) (**4**)

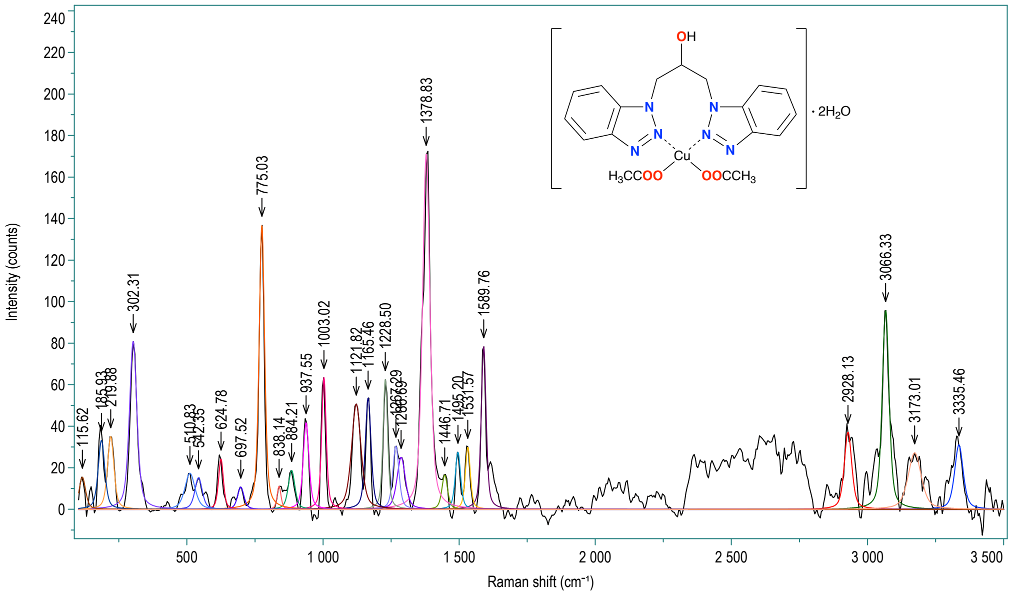

Figure S15. Raman spectrum of diacetate[1,3-bis(benzotriazole-1-yl)propan-2-ol-N,N']copper(II) dihydrated (**5**)

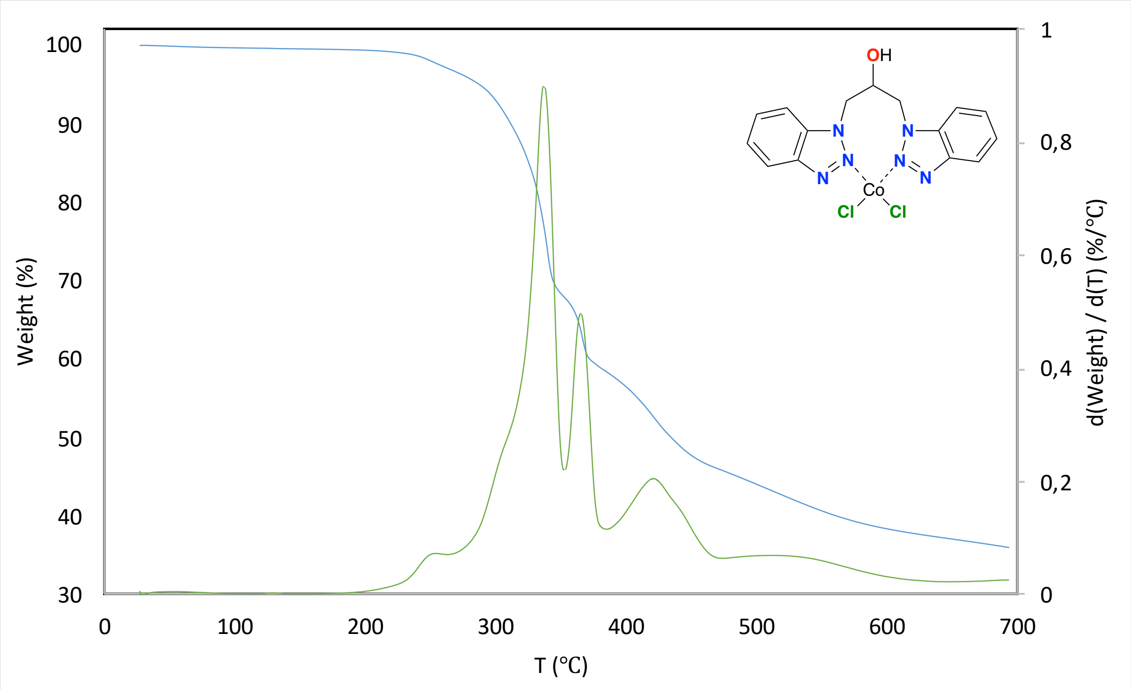

Figure S16. Thermogravimetric (TG) analysis and derivative thermogravimetric (DTG) of dichloro[1,3-bis(benzotriazole-1-yl)propan-2-ol-N,N']cobalt(II) (**2**) in nitrogen atmosphere

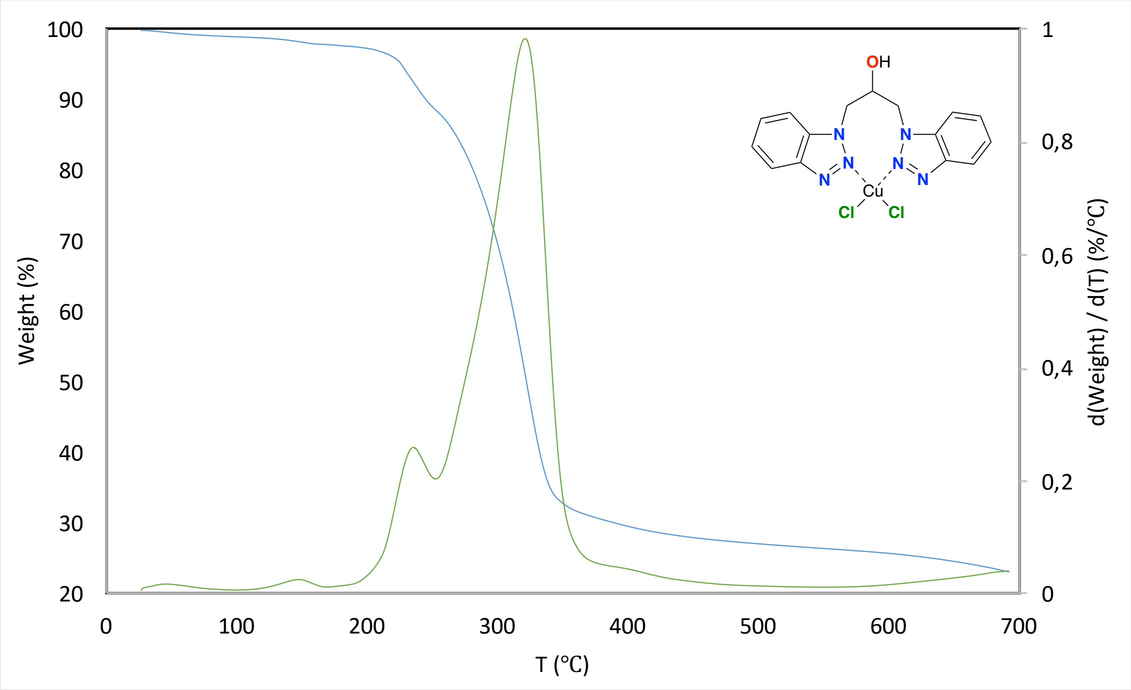

Figure S17. Thermogravimetric (TG) analysis and derivative thermogravimetric (DTG) of dichloro[1,3-bis(benzotriazole-1-yl)propan-2-ol-N,N']copper(II) (**3**) in nitrogen atmosphere

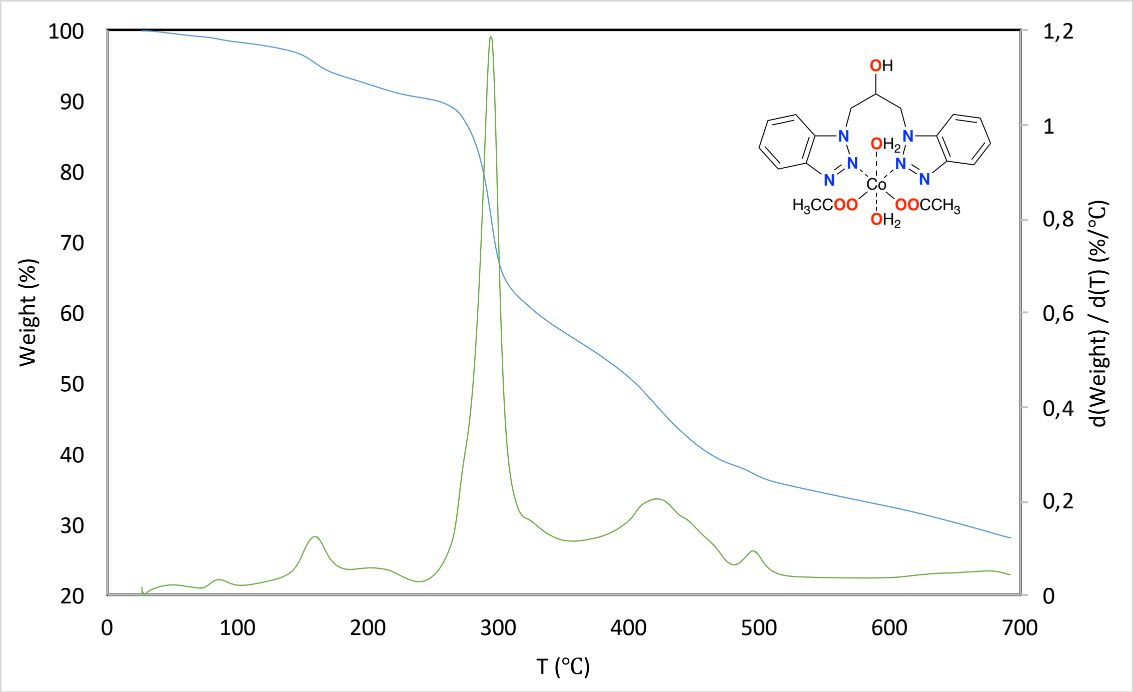

Figure S18. Thermogravimetric (TG) analysis and derivative thermogravimetric (DTG) of diacetate-diaqua-[1,3-bis(benzotriazole-1-yl)propan-2-ol-N,N']cobalt(II) (**4**) in nitrogen atmosphere

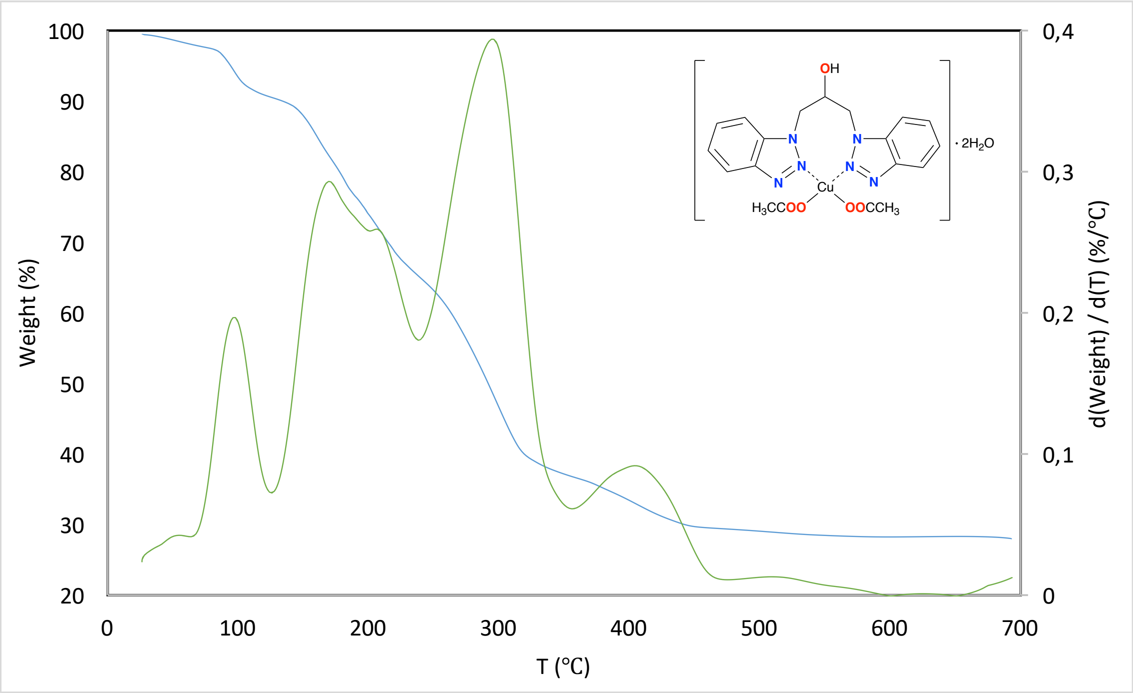

Figure S19. Thermogravimetric (TG) analysis and derivative thermogravimetric (DTG) of diacetate[1,3-bis(benzotriazole-1-yl)propan-2-ol-N,N']copper(II) dihydrated (**5**) in nitrogen atmosphere

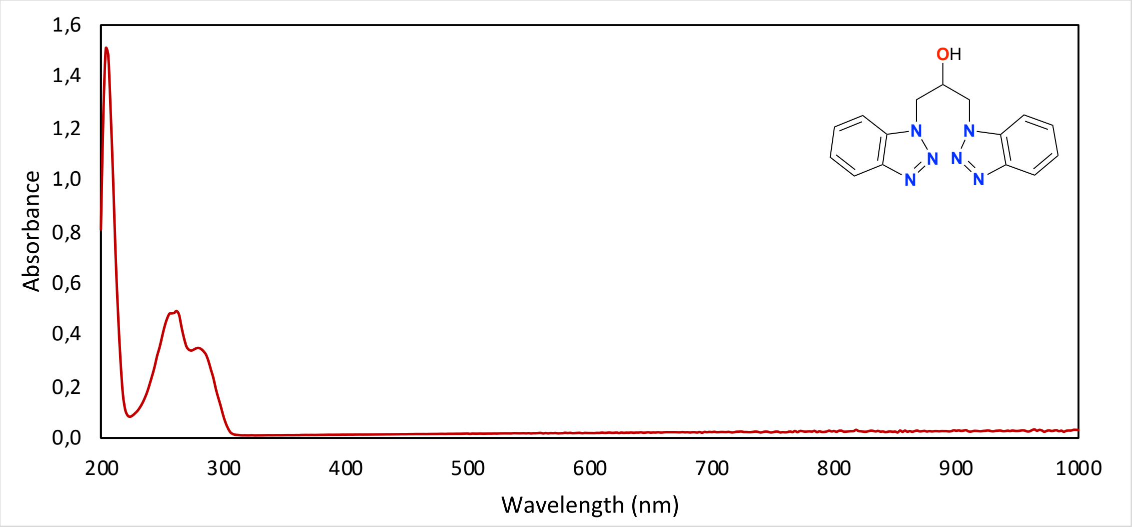

Figure S20. UV-Vis spectrum of 1,3-bis(benzotriazole-1-yl)propan-2-ol (**1**) in methanol. 2.10 x 10^{-5} M, 200-1000 nm region

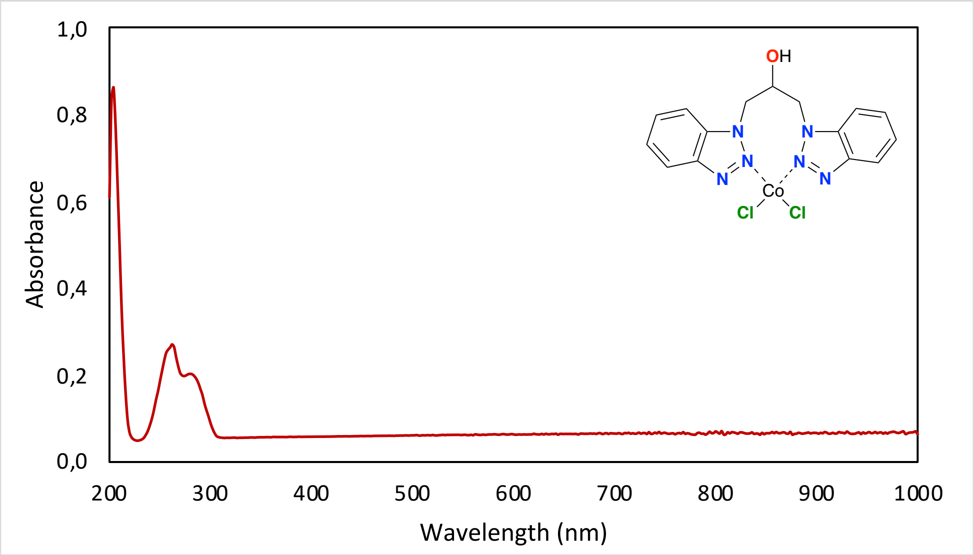

Figure S21. UV-Vis spectrum of dichloro[1,3-bis(benzotriazole-1-yl)propan-2-ol-N,N']cobalt(II) (**2**) in methanol. 2.18 x 10^{-5} M, 200-1000 nm region

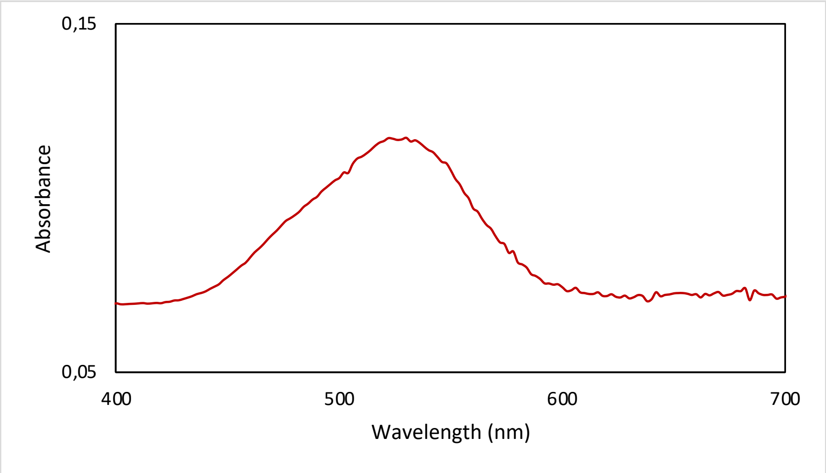

Figure S22. UV-Vis spectrum of dichloro[1,3-bis(benzotriazole-1-yl)propan-2-ol-N,N']cobalt(II) (**2**) in methanol. 4.72 x 10^{-3} M, 400-700 nm region

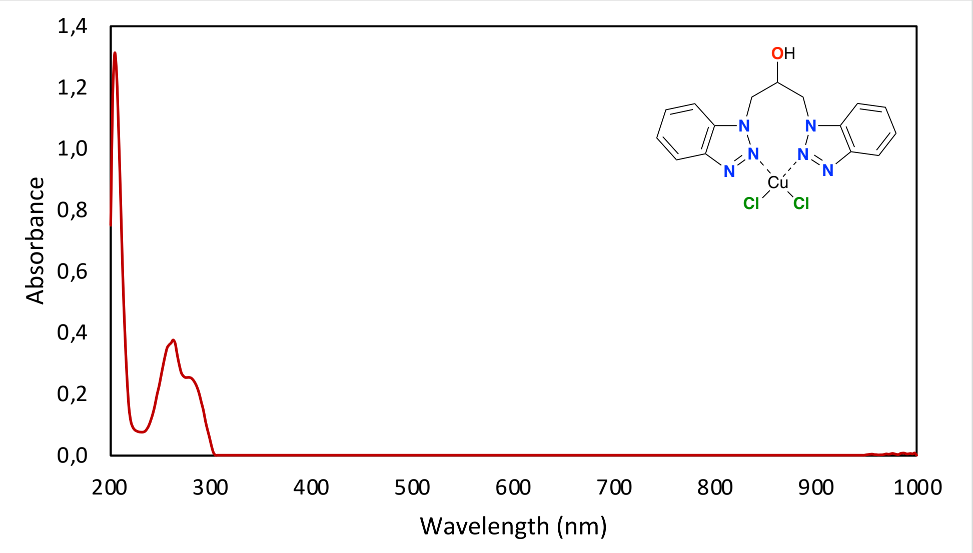

Figure S23. UV-Vis spectrum of dichloro[1,3-bis(benzotriazole-1-yl)propan-2-ol-N,N']copper(II) (**3**) in methanol. 2.16 x 10^{-5} M, 200-1000 nm region

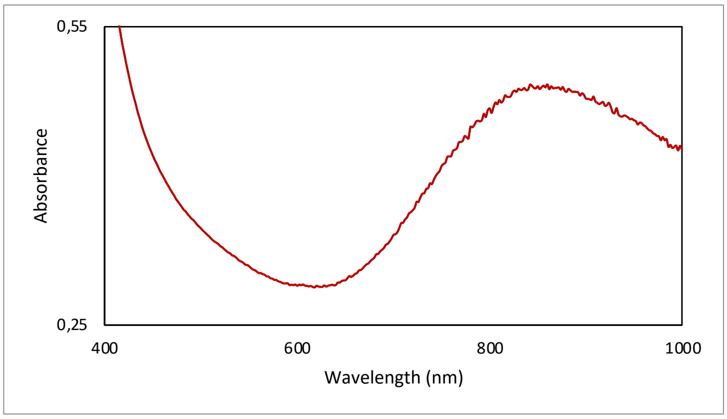

Figure S24. UV-Vis spectrum of dichloro[1,3-bis(benzotriazole-1-yl)propan-2-ol-N,N']copper(II) (**3**) in methanol. 4.66 x 10^{-3} M, 400-1000 nm region

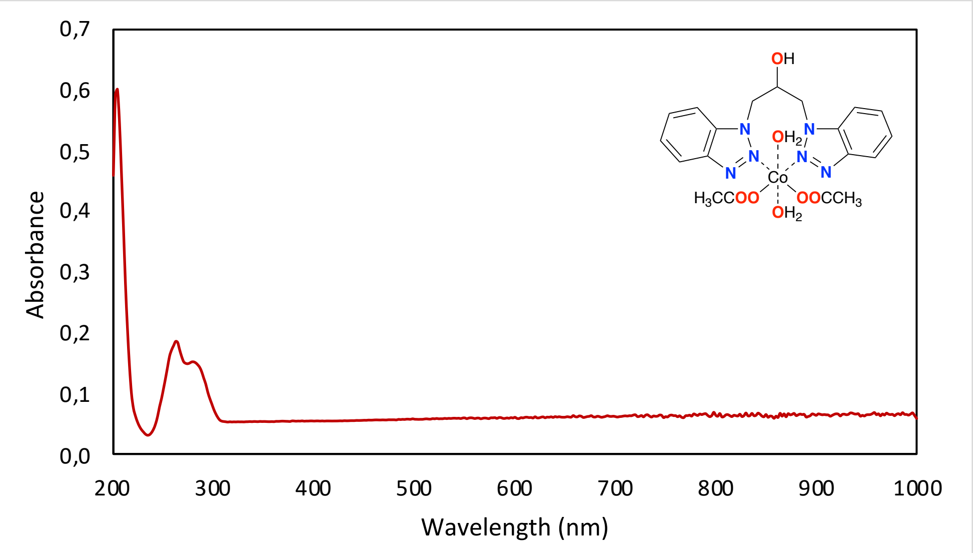

Figure S25. UV-Vis spectrum of diacetate-diaqua-[1,3-bis(benzotriazole-1-yl)propan-2-ol-N,N']cobalt(II) (**4**) in methanol. 1.82 x 10^{-5} M, 200-1000 nm region

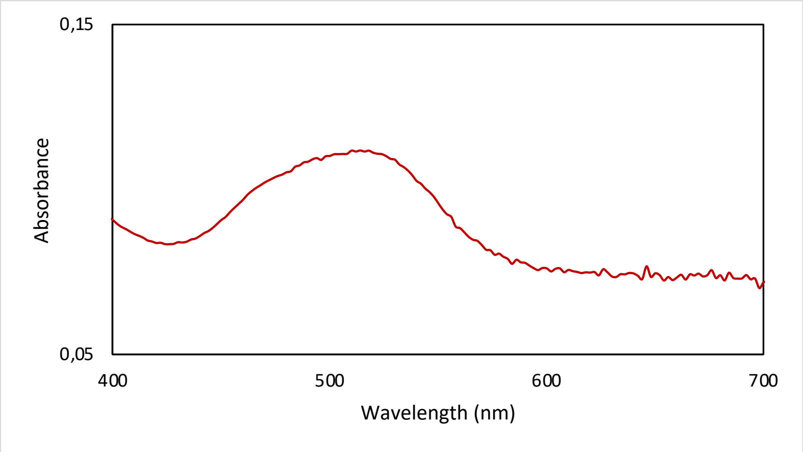

Figure S26. UV-Vis spectrum of diacetate-diaqua-[1,3-bis(benzotriazole-1-yl)propan-2-ol-N,N']cobalt(II) (**4**) in methanol. 3.94 x 10^{-3} M, 400-700 nm region

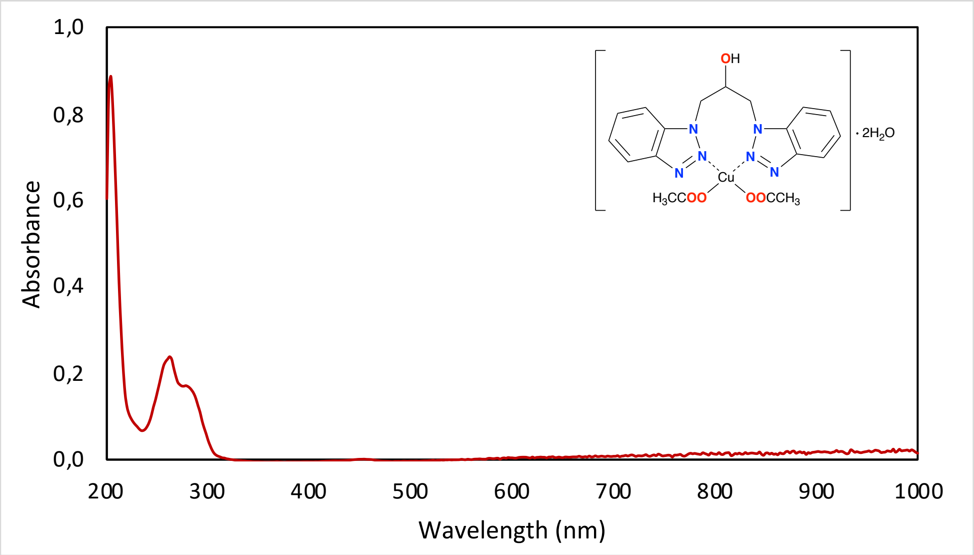

Figure S27. UV-Vis spectrum of diacetate[1,3-bis(benzotriazole-1-yl)propan-2-ol-N,N']copper(II) dihydrated (**5**) in methanol. 1.81 x 10^{-5} M, 200-1000 nm region

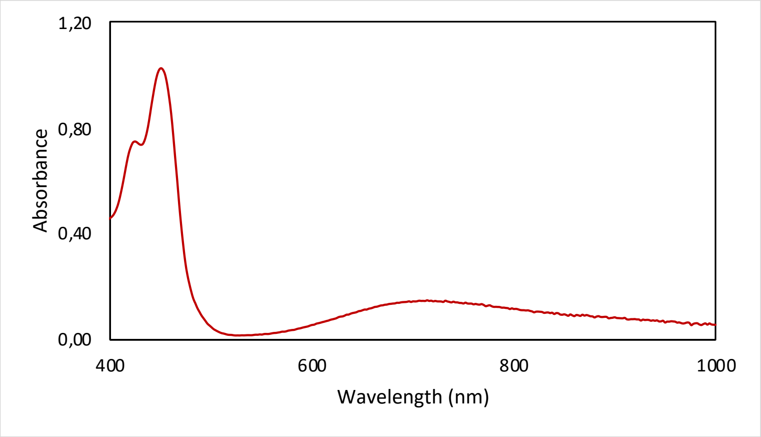

Figure S28. UV-Vis spectrum of diacetate[1,3-bis(benzotriazole-1-yl)propan-2-ol-N,N']copper(II) dihydrated (**5**) in methanol. 3.91 x 10^{-3} M, 400-1000 nm region

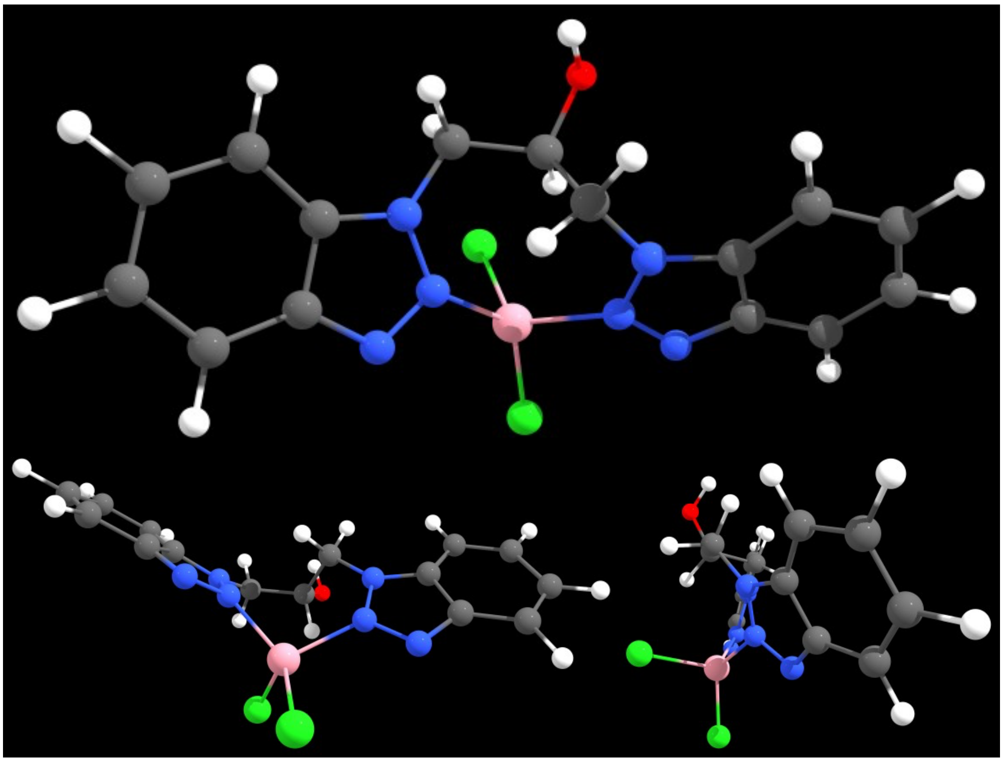

Figure S29. Optimized geometry of conformer Da of (**2**) – Different perspectives

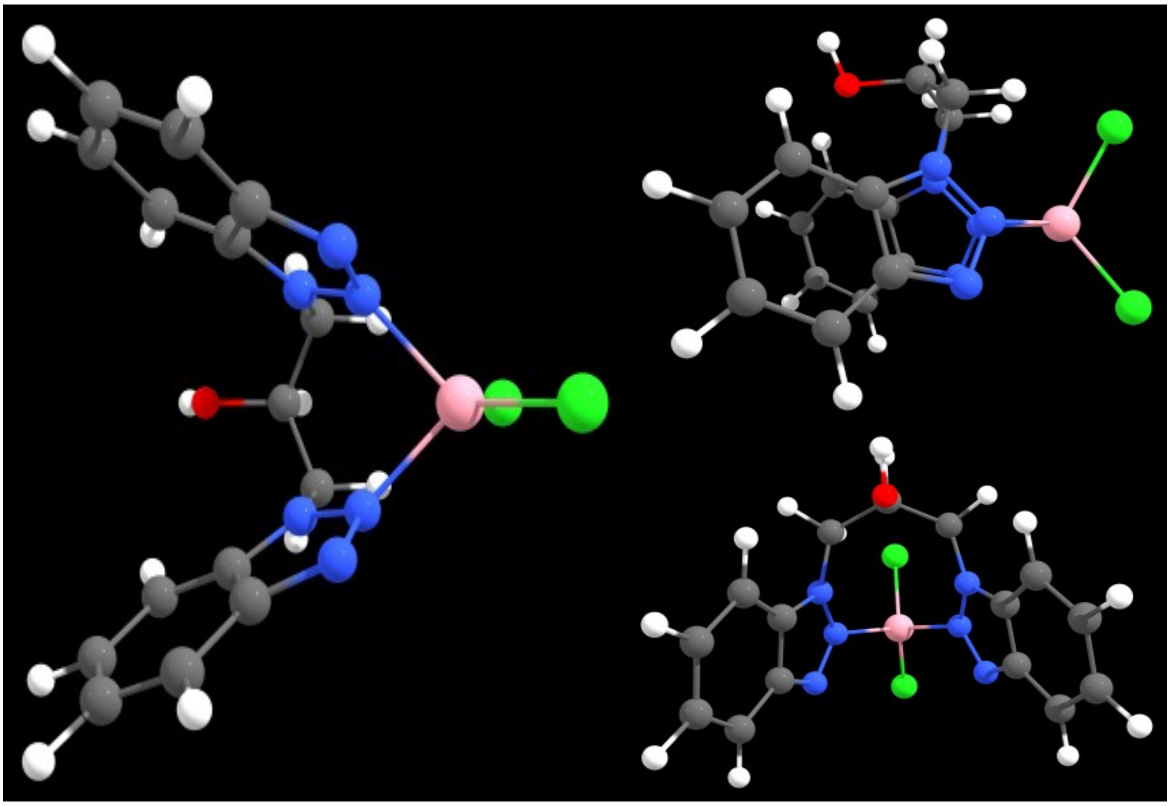

Figure S30. Optimized geometry of conformer Db of (**2**) – Different perspectives

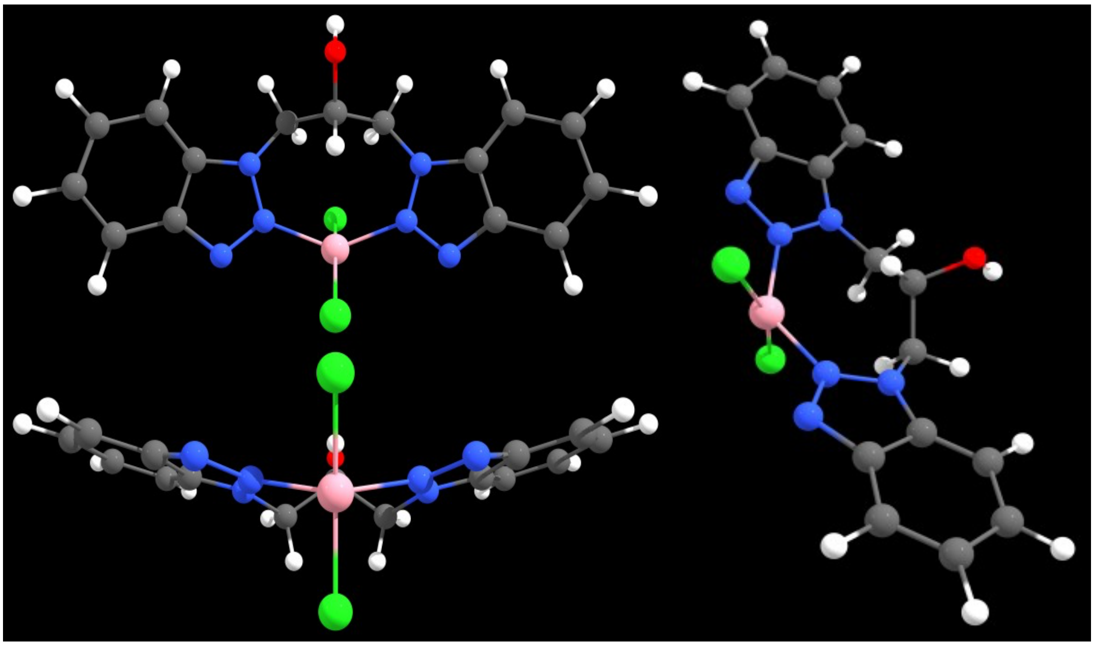

Figure S31. Optimized geometry of conformer Dc of (**2**) – Different perspectives

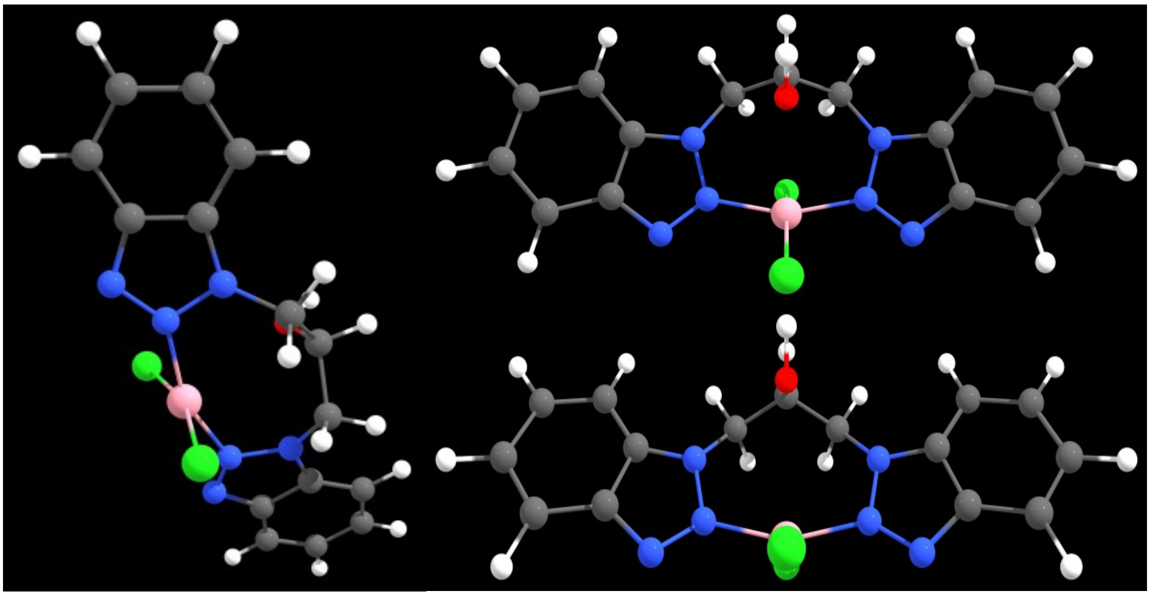

Figure S32. Optimized geometry of conformer Dd of (**2**) – Different perspectives

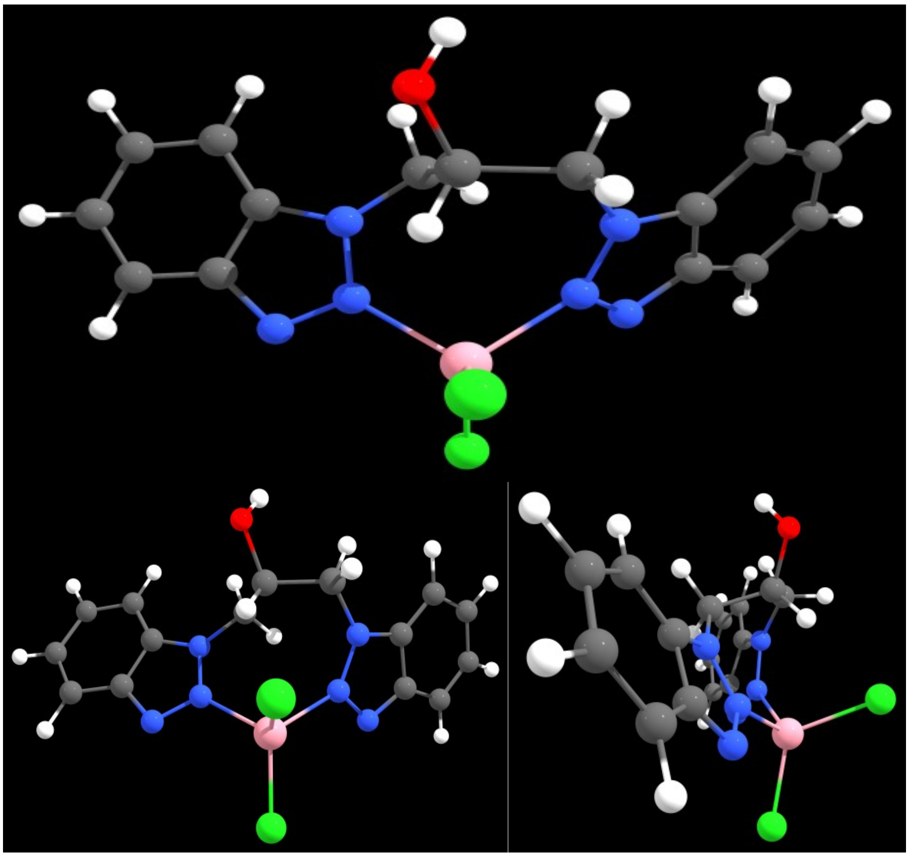

Figure S33. Optimized geometry of conformer Qa of (**2**) – Different perspectives

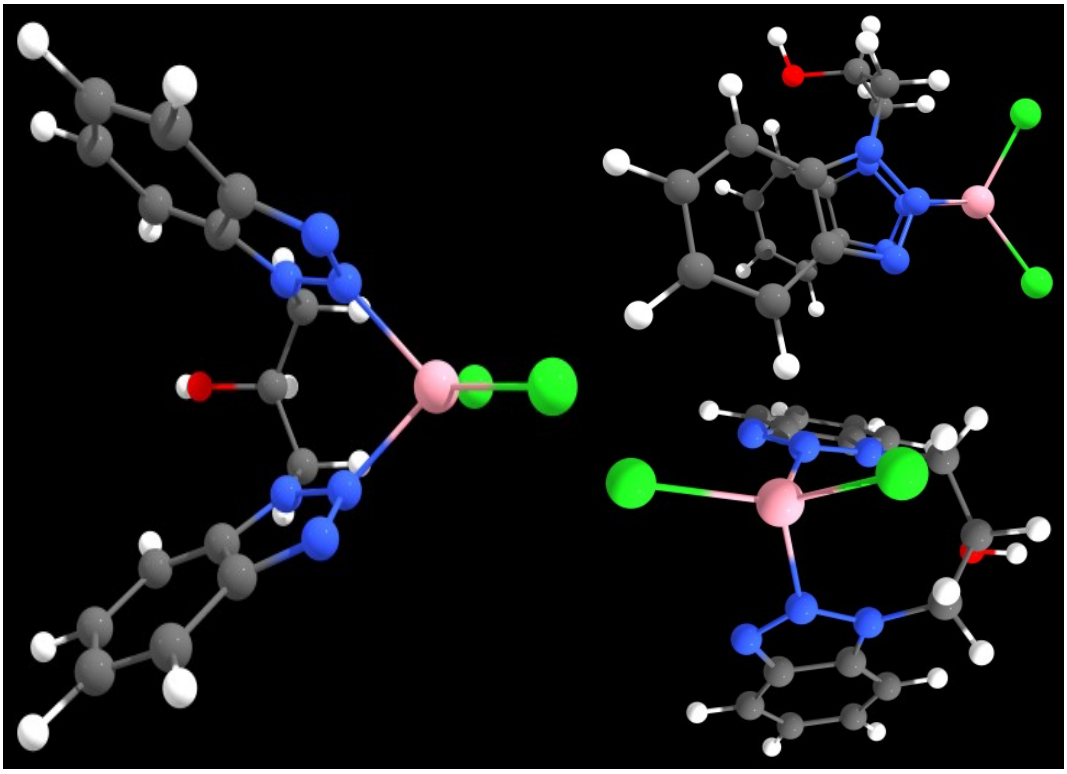

Figure S34. Optimized geometry of conformer Qb of (**2**) – Different perspectives

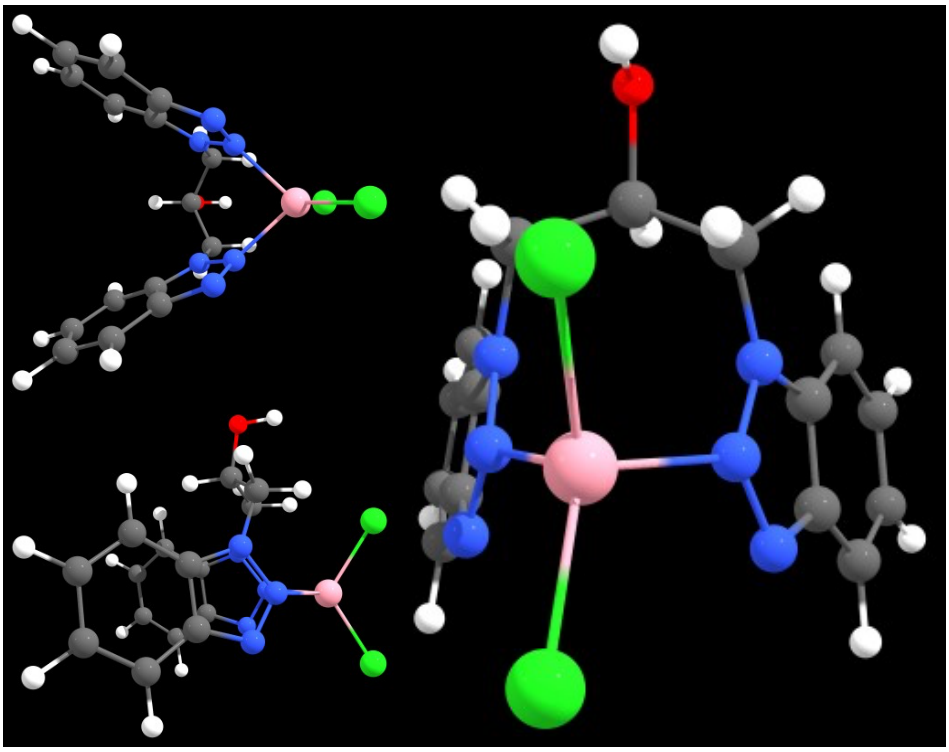

Figure S35. Optimized geometry of conformer Qc of (**2**) – Different perspectives

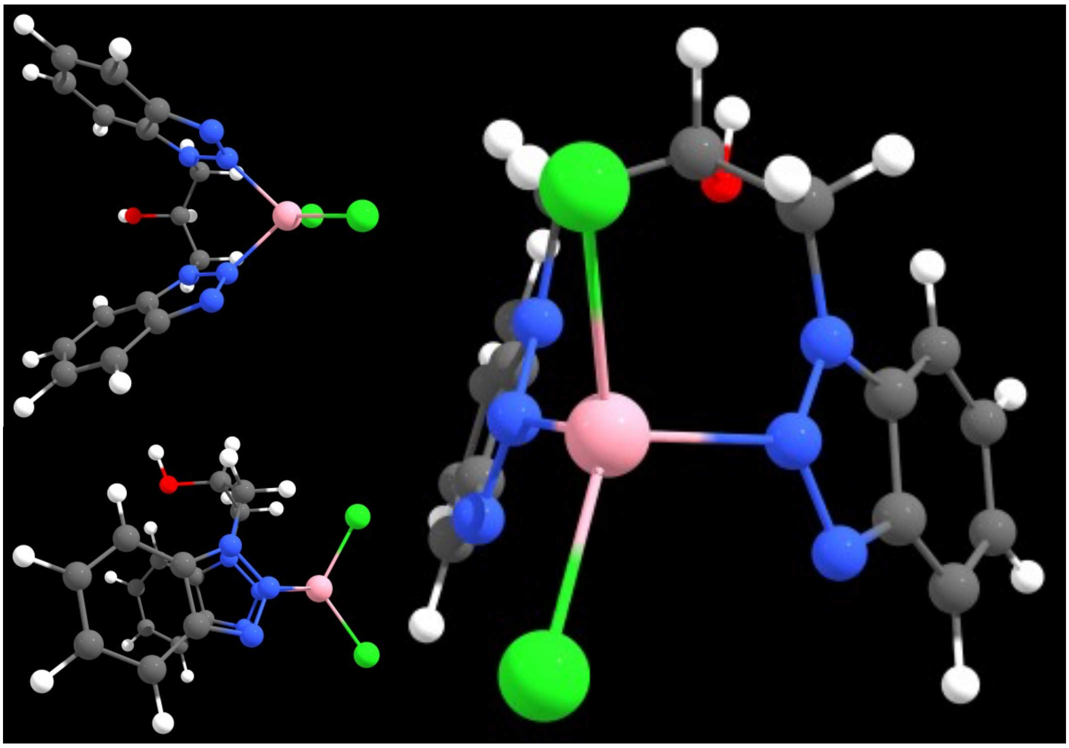

Figure S36. Optimized geometry of conformer Qd of (**2**) – Different perspectives

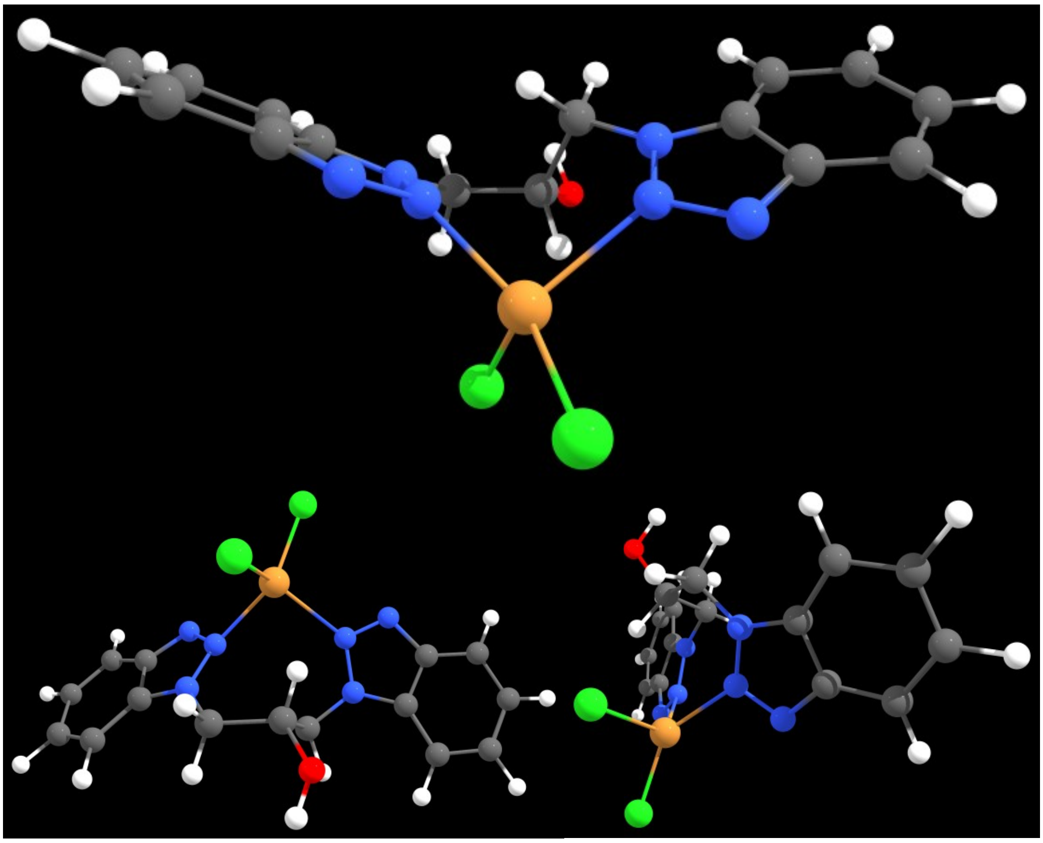

Figure S37. Optimized geometry of conformer Da of (**3**) – Different perspectives

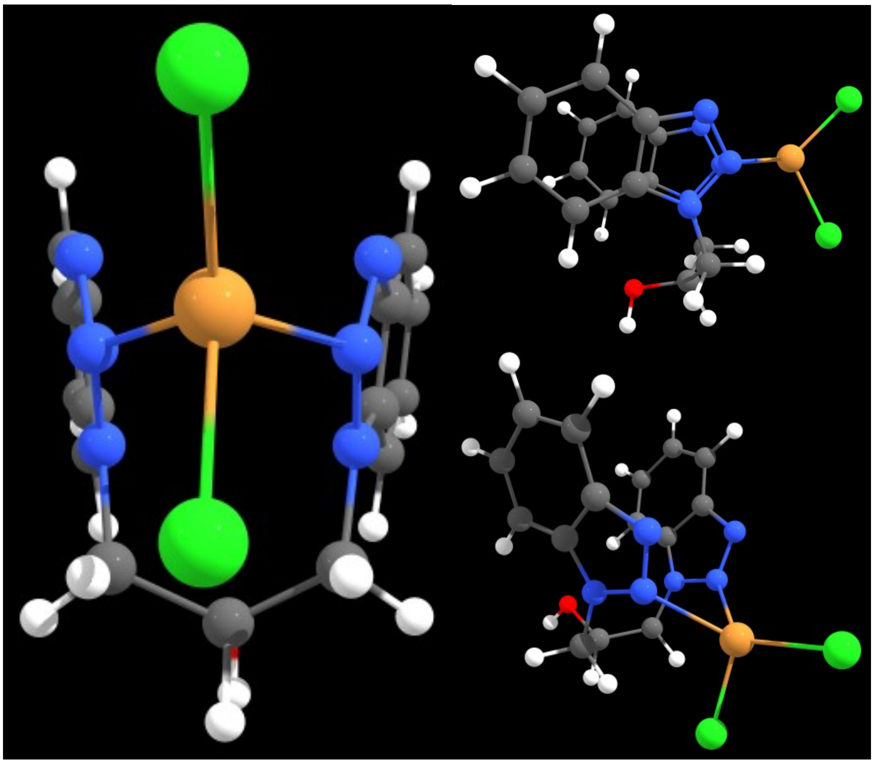

Figure S38. Optimized geometry of conformer Db of (**3**) – Different perspectives

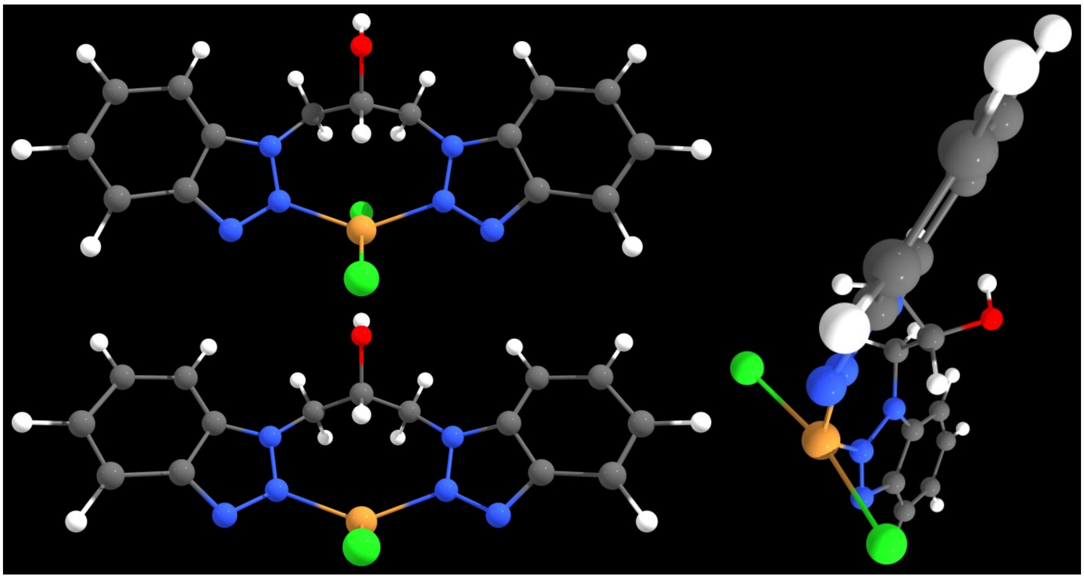

Figure S39. Optimized geometry of conformer Dc of (**3**) – Different perspectives

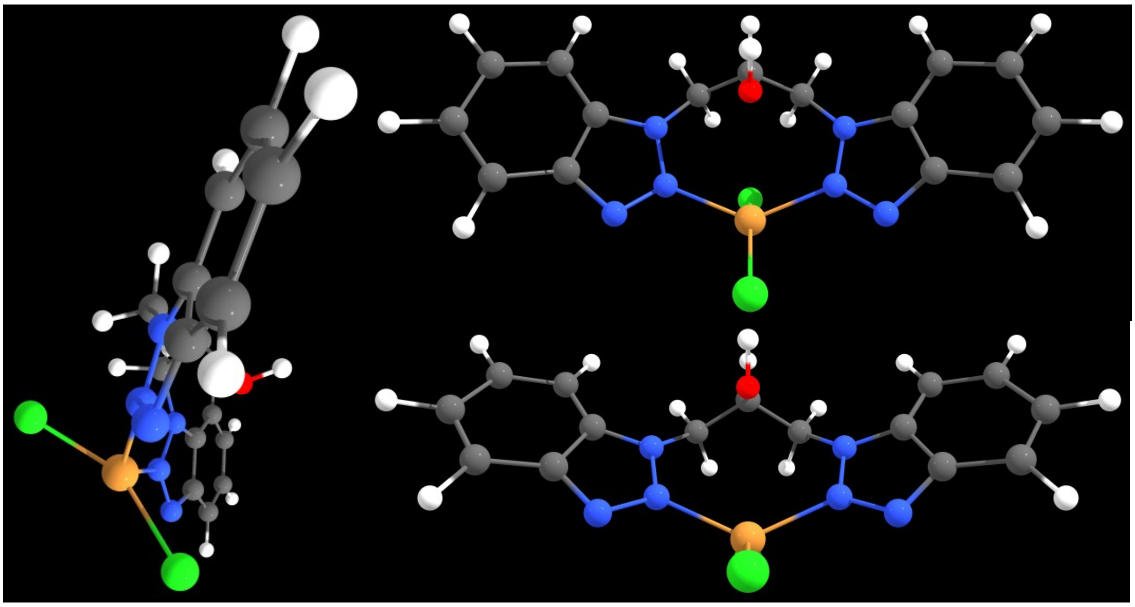

Figure S40. Optimized geometry of conformer Dd of (**3**) – Different perspectives

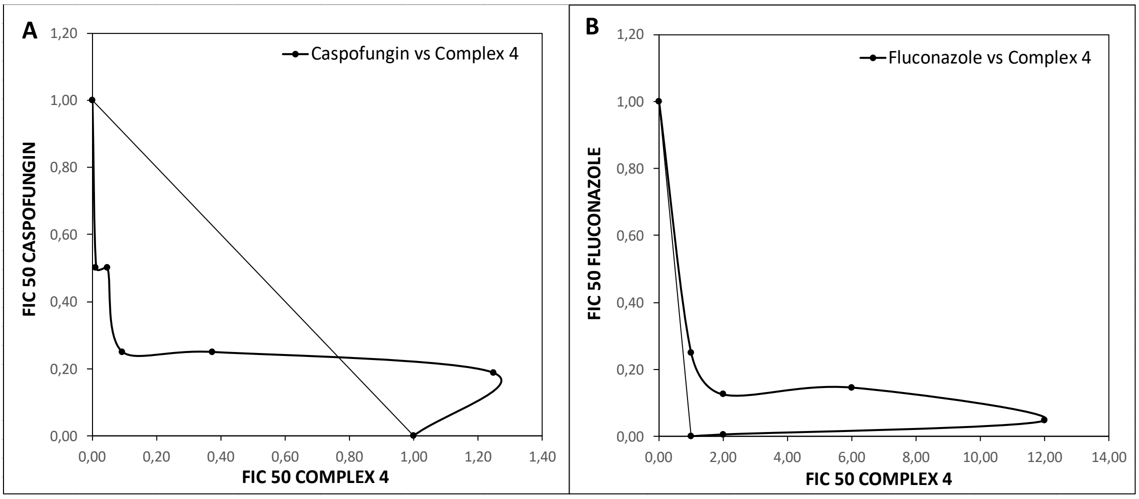

Figure S41. Drug interaction isobolograms. A: A (Caspofungin vs Complex 4 against *C. tropicalis* 66029) B (Fluconazole vs Complex 4 *C. albicans* 90028).

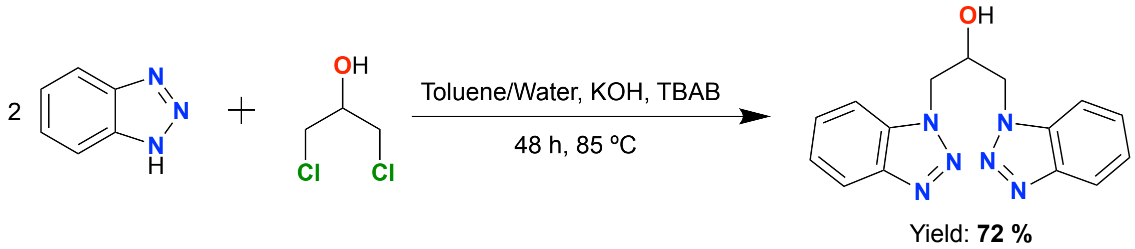

Figure S42. Synthesis of the ligand (**1**). TBAB = Tetrabutylammonium bromide

Table S1. Thermoanalytical results (TGA and DTG) for metal complexes.

Compound (formula)	TG range / °C	DTG_{max}/ °C		Mass loss Estimated (calcd.) / %	Total mass loss Estimated (calcd.) / %	Assignment	Metallic residue
	152-352	254, 337	2	31.63 (31.63)		loss of $C_6H_4N_3 + CH_4$	
**2**	352-385	365	1	9.52 (9.45)	63.55	loss of C_3H_4	$CoCl_2CO$
($C_{15}H_{14}Cl_2CoN_6O$)	385-473	421	1	12.53 (12.74)	(62.78)	loss of CN_3	
	473-694	512	1	9.86 (8.97)		loss of C_3H_2	
	27-170	46, 148	2	1.99 (1.88)		loss of $4H_2$	
**3**	170-253	236	1	9.25 (9.11)	76.79	loss of C_3H_3	CuCl
($C_{15}H_{14}Cl_2CuN_6O$)	253-552	321	1	62.14 (62.41)	(76.91)	loss of $C_{11}N_6ClO$	
	552-694	694	1	3.40 (3.50)		loss of CH_3	
	27-239	88, 159, 202	3	9.38 (9.47)		loss of $2H_2O + C$	
**4**	239-358	294	1	34.10 (34.13)	71.82	loss of $C_6H_4N_3 + C_3H_3O$	CoC_2O_4
($C_{19}H_{24}CoN_6O_7$)	358-573	420, 495	2	22.81 (22.88)	(71.04)	loss of $C_6H_2N_3$	
	573-694	676	1	5.52 (4.55)		loss of $CH_3 + 4H_2$	
	64-126	95	1	7.54 (7.43)		loss of $2H_2O + H_2$	
**5**	126-239	170, 207	2	25.35 (25.42)	70.06	loss of $C_6H_4N_3 + C$	CuC_2O_4
($C_{19}H_{24}CuN_6O_7$)	239-357	296	1	28.37 (28.55)	(70.40)	loss of $C_6H_4N_3 + C_2H_4$	
	357-694	405	1	8.80 (8.99)		loss of C_2H_6O	

Table S2. Antifungal activity of Co(II) and Cu(II) complexes containing 1,3-bis(benzotriazol-1-yl)-propan-2-ol on planktonic cells of *Candida sp*.

MIC: Minimum Inhibitory Concentration; CFM: Minimum fungicidal concentration. Measures expressed in µg mL^{-1}; ND: not determinated. The experiments were performed in triplicate at three independent times.

Compound	*C. albicans* ATCC 90028		*C. albicans* CAP F-13		*C. tropicalis* ATCC 66029		*C. tropicalis* CAP F-01		*C. glabrata* MYA2950		*C. glabrata* CAPF-07		*C. parapsilosis* ATCC22019		*C. parapsilosis* 24754	
	**MIC**	**CFM**	**MIC**	**CFM**	**MIC**	**CFM**	**MIC**	**CFM**	**MIC**	**CFM**	**MIC**	**CFM**	**MIC**	**CFM**	**MIC**	**CFM**
1	>1000	>1000	>1000	>1000	>1000	>1000	>1000	>1000	>400	>400	>1000	>1000	>1000	>1000	>1000	>1000
2	125	250	125	500	125	1000	62.5	500	25	100	62.5	62.5	62.5	125	62.5	500
4	62.5	125	62.5	>1000	62.5	250	62.5	500	15.62	62.5	62.5	500	62.5	250	62.5	500
3	>1000	>1000	>1000	>1000	500	>1000	1000	>1000	>400	>400	>1000	>1000	125	>1000	>1000	>1000
5	>1000	>1000	>1000	>1000	500	>1000	500	>1000	>250	>250	1000	>1000	>1000	>1000	500	>1000
S2	31.25	62.5	31.25	250	31.25	125	7.81	62.5	15.62	31.25	15.6	62.5	31.25	62.5	15.6	125
S4	62.5	125	62.5	>1000	31.25	125	31.25	125	15.65	31.25	15.6	62.5	31.25	125	15.6	62.5
S3	1000	>1000	125	>1000	250	1000	125	1000	>250	>250	125	>1000	>1000	>1000	250	>1000
S5	1000	>1000	125	>1000	125	>1000	125	>1000	>250	>250	250	>1000	>1000	>1000	125	1000
Fluconazole[1]	0.25	>64	32	>64	2.0	>32	32	>64	0.5	0.5	64	>64	1.0	>64	32	>64
Itraconazole[2]	0.03	16	0.5	>16	>16	>16	0.5	>16	0.5	0.5	4	>16	0.0625	4	>16	>16
Caspofungin	0.062	0.125	0.062	0.062	0.125	0.125	1.0	4	ND	ND	ND	ND	ND	ND	ND	ND

Table S3. Matrix of combinations between cobalt(II) complexes and reference drugs (FCZ and Caspofungin).

Combination	Drug A (Complexes)	Drug B (Reference drugs)
1	8X MIC	0
2	4X MIC	1/4 MIC
3	2X MIC	1/2 MIC
4	MIC	MIC
5	1/2 MIC	2X MIC
6	1/4 MIC	4X MIC
7	0	8X MIC
